# Adaptor protein HIP-55-mediated signalosome protects against ferroptosis in myocardial infarction

**DOI:** 10.1038/s41418-022-01110-z

**Published:** 2023-01-13

**Authors:** Yunqi Jiang, Yuhui Qiao, Dan He, Aiju Tian, Zijian Li

**Affiliations:** 1 Department of Cardiology and Institute of Vascular Medicine, Peking University Third Hospital; Beijing Key Laboratory of Cardiovascular Receptors Research; Key Laboratory of Cardiovascular Molecular Biology and Regulatory Peptides, Ministry of Health; Key Laboratory of Molecular Cardiovascular Sciences, Ministry of Education, Beijing, 100191 China; 2 Department of Pharmacy, Peking University Third Hospital, Beijing, 100191 China

**Keywords:** Cardiovascular diseases, Cell biology

## Abstract

Ischemic heart disease is a leading cause of death worldwide. Myocardial infarction (MI) results in cardiac damage due to cell death and insufficient cardiomyocyte self-renewal. Ferroptosis, a novel type of cell death, has recently been shown as a key cause of cardiomyocyte death after MI. However, the complicated regulation mechanisms involved in ferroptosis, especially how ferroptosis is integrated into classical cell survival/death pathways, are still unclear. Here, we discovered that HIP-55, a novel adaptor protein, acts as a hub protein for the integration of the ferroptosis mechanism into the classical AKT cell survival and MAP4K1 cell death pathways for MI injury. The expression of HIP-55 is induced in MI. Genetic deletion of HIP-55 increased cardiomyocyte ferroptosis and MI injury, whereas cardiac-specific overexpression of HIP-55 significantly alleviated cardiomyocyte ferroptosis and MI injury. Mechanistically, HIP-55 was identified as a new AKT substrate. AKT phosphorylates HIP-55 at S269/T291 sites and further HIP-55 directs AKT signaling to negatively regulate the MAP4K1 pathway against MI injury in a site-specific manner. S269A/T291A-mutated HIP-55 (HIP-55AA), which is defective in AKT phosphorylation and significantly decreases the interaction between HIP-55 and MAP4K1, failed to inhibit the MAP4K1/GPX4 ferroptosis pathway. In line with this mechanism, cardiac-specific overexpression of HIP-55WT mice, but not cardiac-specific overexpression of HIP-55AA mice, protected cardiomyocytes against MI-induced ferroptosis and cardiac injury in vivo. These findings suggest that HIP-55 rewired the classical AKT (cell survival) and MAPK (cell death) pathways into ferroptosis mechanism in MI injury. HIP-55 may be a new therapeutic target for myocardial damage.

## Introduction

Myocardial infarction (MI) and subsequent heart failure (HF) are the leading causes of death worldwide [1]. The main pathophysiology basis of MI is cardiomyocyte death due to lack of blood perfusion. Because adult cardiomyocytes are terminally differentiated, they are incapable of sufficient regeneration and proliferation [2]. Although recent evidence suggest that the cardiac stem cells may contribute to cardiomyocyte renewal, it is totally insufficient for myocardial repair after MI. Therefore, the extent of cell death is the determining factor for infarct size and clinical outcomes. Preventing cardiac cell death may be an effective cardioprotective strategy; however, preventive measures have yet to be developed, mainly due to the elusive mechanisms underlying MI-induced cell death.

For some time, apoptosis has been considered the main form of myocardial cell death, but there is accumulating evidence that ferroptosis is also a major contributor to cardiomyocyte death in various heart diseases, including HF, ischemic heart disease, and cardiomyopathy, among others [3]. Ferroptosis inducers, including ferric iron, erastin, and Ras-selective lethal 3 (RSL3), obviously induce cardiomyocyte death [4]. While ferroptosis inhibitor, ferrostatin-1, or iron chelators, can reduce cardiac infarction size and injury significantly, establishing the crucial role of ferroptosis in MI-induced cardiomyocyte death [5, 6]. Clinical studies have also demonstrated that iron overload exists in ST-segment-elevation MI and associated with adverse cardiac remodeling [7]. Taken together, these researches indicated that ferroptosis plays a crucial role in cardiomyocyte death. Ferroptosis as one of a number of cell death mechanisms is regulated by specific pathways and is involved in diverse biological contexts. However, the complicated regulation mechanisms of ferroptosis, and how it is integrated into classical cell survival/death pathways are still unclear.

Here, we showed that HIP-55, an adaptor protein, acts as a hub protein for the integration of the ferroptosis mechanism into the classical AKT cell survival and MAP4K1 cell death pathways for MI injury. HIP-55 significantly alleviated ferroptosis and MI injury. Mechanistically, HIP-55 was identified as a new AKT substrate, phosphorylated at S269/T291. After phosphorylation, HIP-55 negatively regulates the MAP4K1-dependent JNK/GPX4 ferroptosis pathway against MI injury. Our findings revealed that adaptor HIP-55 functions as a nodal control point for cardiomyocyte ferroptosis that coordinates the dynamic responses of the AKT cell survival and MAP4K1-dependent JNK/GPX4 ferroptosis pathway in MI.

## Results

### Characterization of myocardial HIP-55 expression

**Fig. 1:**
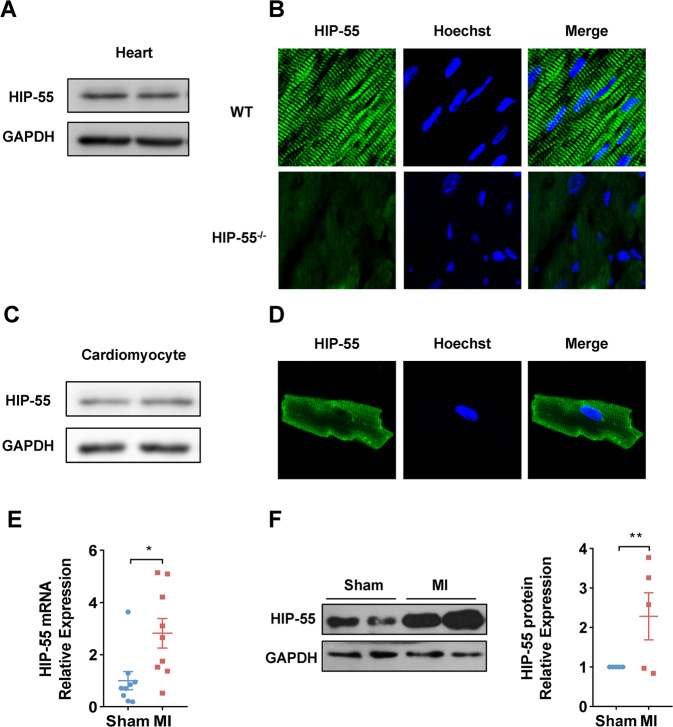
Characterization of myocardial HIP-55 expression. **A** Immunoblot detected expression of HIP-55 in mouse heart tissue. **B** Immunofluorescence detected expression of HIP-55 in the wildtype (WT) and HIP-55 knockout (HIP-55^−/−^) mouse heart tissue. **C** Immunoblot and **D** immunofluorescence detected expression of HIP-55 in the isolated primary cardiomyocytes. **E**, **F** Detection of HIP-55 mRNA (**E**) and protein levels (**F**) in the heart tissues of sham-operated mice and myocardial infarcted mice. *n* = 9 in **E**, *n* = 5 in **F**. *P < 0.05, **P < 0.01. All error bars represent mean ± SEM.

To identify a new regulatory mechanism of MI, comparative microarray analysis was previously performed to reveal genes associated with MI. HIP-55 was found to be markedly higher in the hearts of MI mice than that in control mice, suggesting that HIP-55 could be a novel regulator of MI. To further confirm the differentially expressed HIP-55 discovered by microarray, cardiac expression of HIP-55 was characterized. We confirmed the expression of HIP-55 in heart tissue (Fig. 1A, B) and cardiomyocytes (Fig. 1C, D) by western blotting and immunofluorescence, respectively. HIP-55 expression was significantly upregulated in the infarcted hearts of MI mice both at mRNA (Fig. 1E) and protein level (Fig. 1F), which indicated a potential role for HIP-55 in MI.

### Genetic deletion of HIP-55 increases cardiomyocyte injury after MI

**Fig. 2:**
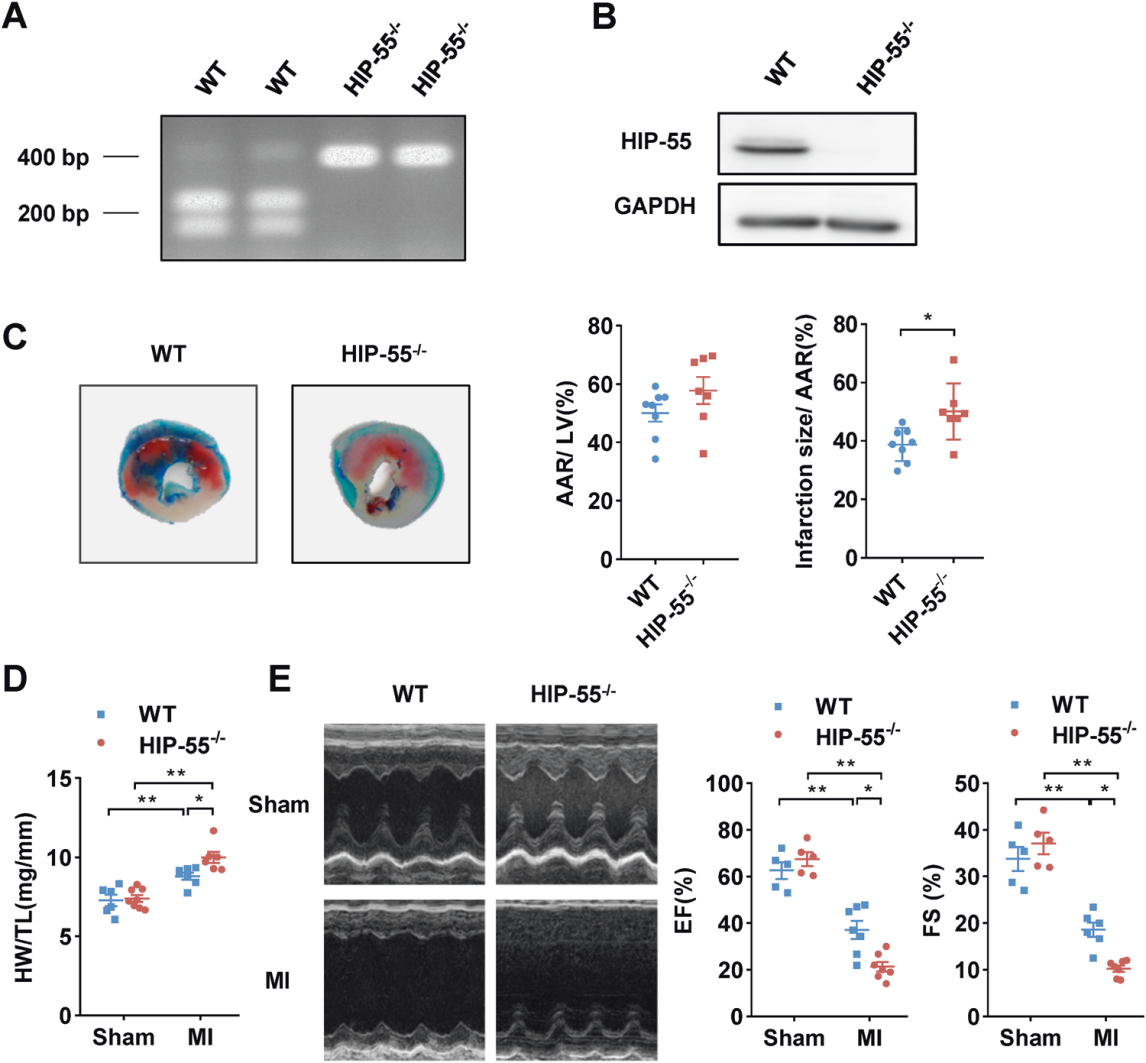
Genetic deletion of HIP-55 increases cardiomyocyte injury after MI. **A** Genotyping of wildtype (WT) and HIP-55 knockout (HIP-55^−/−^) mice. **B** Western blot analysis of HIP-55 expression in the heart tissues of WT and HIP-55^−/−^ mice. **C** Deletion of HIP-55 promoted myocardial infarction size. Representative Alcian blue-TTC staining (left) and quantitative analysis (right) for area at risk (AAR) and infarct size in hearts from the WT and HIP-55^−/−^ mice post-MI. WT, *n* = 8; HIP-55^−/−^, *n* = 7. **D** HIP-55 deficiency significantly increased cardiac hypertrophy after MI. The ratio of heart weight to tibial length (HW/TL) in WT and HIP-55^−/−^ mice after MI. *n* = 6–8. **E** Deletion of HIP-55 promoted MI-induced cardiac dysfunction. Representative M-mode echocardiographic photographs (left) and cardiac contractile function (right) quantified by echocardiographic analysis of ejection fraction (EF) and fractional shortening (FS) in WT and HIP-55^−/−^ mice at day 7 after MI. *n* = 5–7. *P < 0.05, **P < 0.01. All error bars represent mean ± SEM.

To elucidate the biological functions of HIP-55 in MI, HIP-55 knockout (HIP-55^−/−^) mice were generated and characterized (Fig. 2A, B). HIP-55^−/−^ mice or control wildtype (WT) mice were subjected to left arterial descending artery ligation to induce MI. Alcian blue-triphenyltetrazolium chloride (TTC) staining demonstrated increased infarction size in HIP-55 knockout mice compared with control mice (Fig. 2C). Heart weight/tibia length (HW/TL) revealed HIP-55 deficiency also significantly increased cardiac hypertrophy after MI (Fig. 2D). Furthermore, echocardiography analysis indicated that MI-induced cardiac contractile dysfunction was markedly increased in HIP-55^−/−^ mice (Fig. 2E), indicating that HIP-55 deficiency facilitated adverse cardiac remodeling after MI. These results revealed that the presence of HIP-55 protects against cardiac injury following MI.

### Cardiac-specific overexpression of HIP-55 alleviates MI injury

**Fig. 3:**
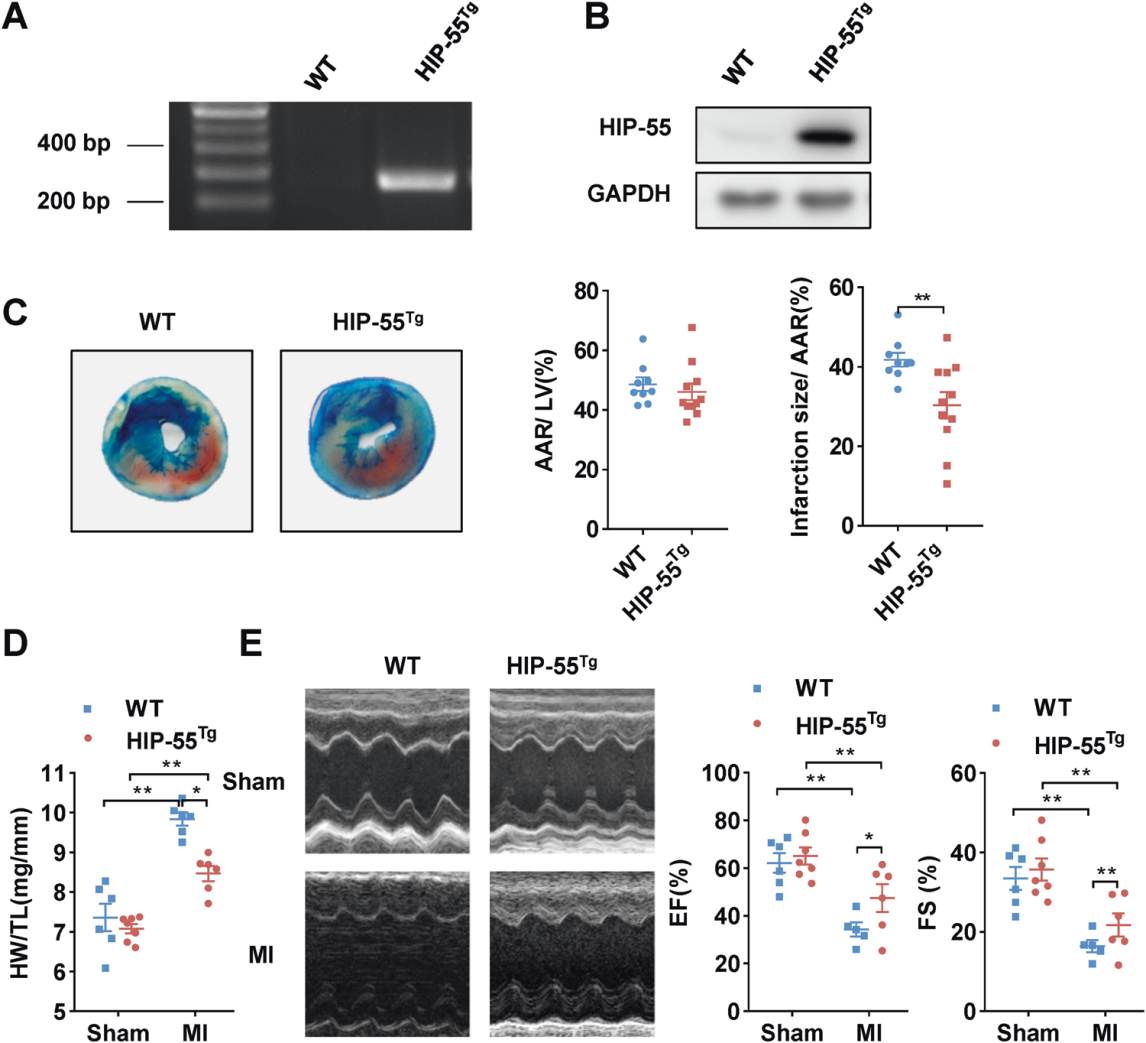
Cardiac-specific overexpression of HIP-55 alleviates MI injury. **A** Genotyping of WT and cardiac-specific overexpression of HIP-55 (HIP-55^Tg^) mice. **B** Western blot analysis of HIP-55 expression in the heart tissues of WT and HIP-55^Tg^mice. **C** Cardiac-specific overexpression of HIP-55 suppressed myocardial infarction size. Representative Alcian blue-TTC staining of the heart tissue obtained from the WT and HIP-55^Tg^ mice post-MI (left). Quantification of the ratios of AAR/LV and infarct size of each group (right). WT, *n* = 9; HIP-55^Tg^, *n* = 11. **D** The ratio of heart weight to tibial length (HW/TL) in WT and HIP-55^Tg^ mice after MI. *n* = 6–7. **E** Cardiac-specific overexpression of HIP-55 suppressed MI-induced cardiac dysfunction. Representative M-mode echocardiographic photographs (left) and cardiac contractile function (right) quantified by echocardiographic analysis of ejection fraction (EF) and fractional shortening (FS) in WT and HIP-55^Tg^ mice at day 7 after MI. *n* = 5–7. *P < 0.05, **P < 0.01. All error bars represent mean ± SEM.

To further clarify the cardiac-specific role of HIP-55, we generated cardiac-specific overexpression of HIP-55 transgenic mice (HIP-55^Tg^) (Fig. 3A, B). The HIP-55^Tg^ and control mice underwent induced MI as before. Compared with control mice, cardiac-specific overexpression of HIP-55 mice showed obviously decreased infarction size (Fig. 3C). Furthermore, cardiac-specific overexpression of HIP-55 markedly inhibited cardiac hypertrophy after MI (Fig. 3D). Echocardiography analysis also revealed that HIP-55^Tg^ mice preserved better cardiac function than control mice (Fig. 3E). In brief, these results indicated that cardiac-specific overexpression of HIP-55 protected cardiomyocytes against the MI injury.

### HIP-55 inhibits MI-induced ferroptosis

**Fig. 4:**
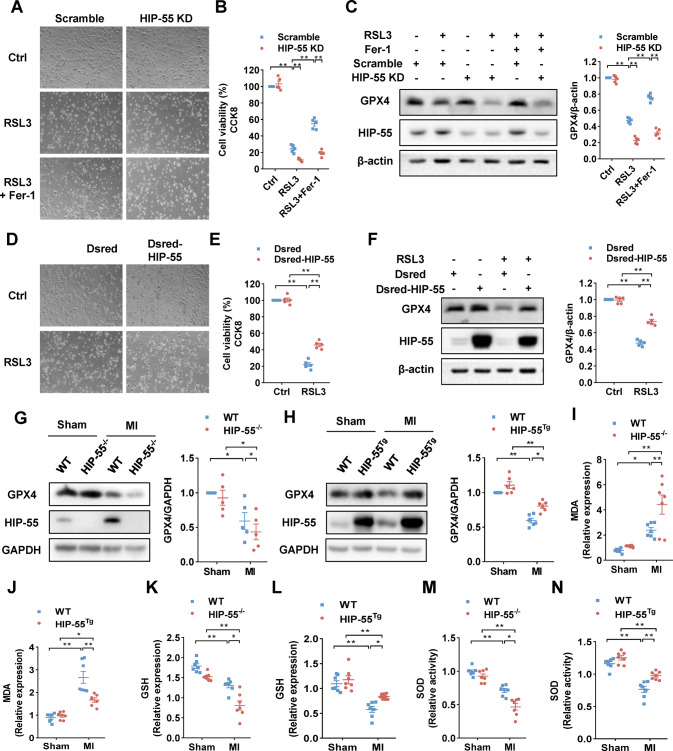
HIP-55 inhibits MI-induced ferroptosis. **A** HIP-55 inhibited RSL3-induced cardiomyocyte ferroptosis. Cardiomyocytes were transfected as indicated and then treated with RSL3 (5 μM), or ferrostatin-1 (Fer-1, 20 μM) for 24 h and then imaged (magnification, ×10). *n* = 5. **B** Cell viability was determined by CCK-8 kit. *n* = 5. **C** Cardiomyocytes were transfected as indicated and then treated with RSL3 (5 μM), or ferrostatin-1 (Fer-1, 20 μM) for 16 h, and then expression of GPX4 detected. *n* = 5. **D** Cardiomyocytes were transfected as indicated and then treated with RSL3 (5 μM) for 24 h, and then imaged (magnification, ×10). *n* = 5. **E** Cell viability was determined by CCK-8 kit. *n* = 5. **F** Cardiomyocytes were transfected as indicated and then treated with RSL3 (5 μM) for 16 h, and then expression of GPX4 detected. *n* = 5. **G** Deficiency of HIP-55 significantly worsened MI-induced GPX4 downregulation. Representative immunoblots and statistical analysis of GPX4 expression in WT and HIP-55^−/−^ mouse hearts after MI. *n* = 5. **H** Cardiac-specific overexpression of HIP-55 alleviated MI-induced GPX4 downregulation. Representative immunoblots and statistical analysis of GPX4 expression in WT and HIP-55^Tg^ mice hearts after MI. *n* = 6. **I** Relative expression of MDA in the heart tissues of WT and HIP-55^−/−^ mice after MI. *n* = 7. **J** Relative expression of MDA in heart tissue of WT and HIP-55^Tg^ mice after MI. *n* = 7. **K** Relative expression of GSH in heart tissue of WT and HIP-55^−/−^ mice after MI. *n* = 7. **L** Relative expression of GSH in heart tissue of WT and HIP-55^Tg^ mice after MI. *n* = 7. **M** Relative activity of SOD in heart tissue of WT and HIP-55^−/−^ mice after MI. *n* = 7. **N** Relative activity of SOD in heart tissue of WT and HIP-55^Tg^ mice after MI. *n* = 7. *P < 0.05, **P < 0.01. All error bars represent mean ± SEM.

Ferroptosis has been shown to play an important role in cardiomyocyte death in MI [5, 6]. We first explored the role of HIP-55 using the specific ferroptosis inducer RSL3 in cardiomyocytes. Knockdown of HIP-55 aggravated RSL3-induced cardiomyocyte ferroptosis (Fig. 4A, B) and downregulated glutathione peroxidase (GPX4) protein, a key regulator of ferroptosis (Fig. 4C). Furthermore, we observed that knockdown of HIP-55 weakened the inhibition of ferroptosis by ferrostatin-1 (Fer-1) (Fig. 4A–C). Overexpression of HIP-55 prevented RSL3-induced cardiomyocyte ferroptosis (Fig. 4D–F). These studies demonstrated the important protective role against HIP-55 in cardiomyocyte ferroptosis.

In MI, our results showed that deficiency of HIP-55 significantly decreased the expression of GPX4 protein (Fig. 4G), while cardiac-specific overexpression of HIP-55 markedly promoted its expression (Fig. 4H). Furthermore, we detected a role for HIP-55 in lipid peroxidation and redox state, the hallmarks of ferroptosis [8]. Analysis of malondialdehyde (MDA), a lipid peroxidation marker, showed that deficiency of HIP-55 aggravated lipid peroxidation after MI (Fig. 4I), whereas cardiac-specific overexpression of HIP-55 decreased peroxidation (Fig. 4J). Glutathione (GSH) and superoxide (SOD) content detection also revealed that deficiency of HIP-55 worsened MI-induced oxidative stress (Fig. 4K, L), while overexpression of HIP-55 alleviated it (Fig. 4M, N). Taken together, these results indicate that HIP-55 inhibits ferroptosis in MI.

### HIP-55 inhibits the MAP4K1-dependent JNK/GPX4 ferroptosis pathway

**Fig. 5:**
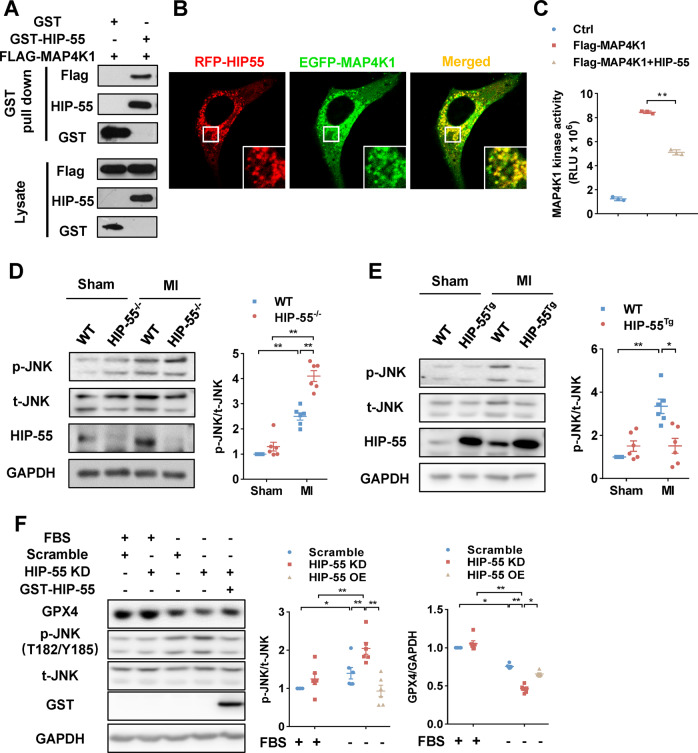
HIP-55 inhibits the MAP4K1-dependent JNK/GPX4 ferroptosis pathway. **A** GST pull-down confirmed HIP-55 interaction with MAP4K1. HEK293A cells were co-transfected with GST or GST-HIP-55 with Flag-MAP4K1 plasmids. Cell lysates were immunoprecipitated with Glutathione-Sepharose 4B beads and then subjected to immunoblotting. *n* = 6. **B** Co-localization between HIP-55 and MAP4K1. NIH-3T3 cells were co-transfected with indicated plasmids for confocal photography. *n* = 6. **C** HIP-55 inhibited MAP4K1 kinase activity. Flag-tagged MAP4K1 was co-transfected with GST or GST-HIP-55 plasmids in HEK293A cells. MAP4K1 was purified with anti-FLAG beads to detect kinase activity by using an ADP-Glo™ Kinase Assay. *n* = 3. **D** Deficiency of HIP-55 significantly promoted MI-induced JNK over-activation. Representative immunoblots and statistical data of JNK activation in WT and HIP-55^−/−^ mice hearts after MI. *n* = 5. **E** Cardiac-specific overexpression of HIP-55 suppressed MI-induced JNK over-activation. Representative immunoblots and statistical data on JNK activation in WT and HIP-55^Tg^ mice hearts after MI. *n* = 5. **F** HIP-55 inhibited the JNK/GPX4 ferroptosis pathway. Cells were transfected with plasmids as indicated and then subjected to starvation for 24 h. Then the expression of GPX4 and phosphorylation of JNK were then detected. *n* = 6. *P < 0.05, **P < 0.01. All error bars represent mean ± SEM.

MAPK cascade signaling plays an important role in various cell death pathways. MAP4K1, the most proximal protein kinase in the MAPK superfamily, specifically initiates the JNK MAPK cascade to induce cell death, including in ferroptosis and apoptosis [9–11]. MAP4K1 contains a proline-rich domain, which could bind with the SH3 domain of HIP-55. Both GST pull-down and confocal co-localization analyses showed that HIP-55 interacted with MAP4K1 (Fig. 5A, B). Furthermore, the kinase activity assay showed that HIP-55 decreased MAP4K1 kinase activity (Fig. 5C). Accordingly, deficiency of HIP-55 significantly increased MAP4K1 downstream kinase JNK phosphorylation induced by MI injury (Fig. 5D). In contrast, cardiac-specific overexpression of HIP-55 remarkably decreased MI-induced activation of JNK (Fig. 5E). Moreover, HIP-55 inhibited the JNK/GPX4 ferroptosis pathway, since knockdown of HIP-55 increased JNK phosphorylation and decreased expression of GPX4, which could be reversed by overexpression of HIP-55 (Fig. 5F). These results indicated that HIP-55 inhibited MI-induced ferroptosis by negatively regulating the MAP4K1/JNK/GPX4 pathway.

### HIP-55 is a new substrate for AKT kinase

**Fig. 6:**
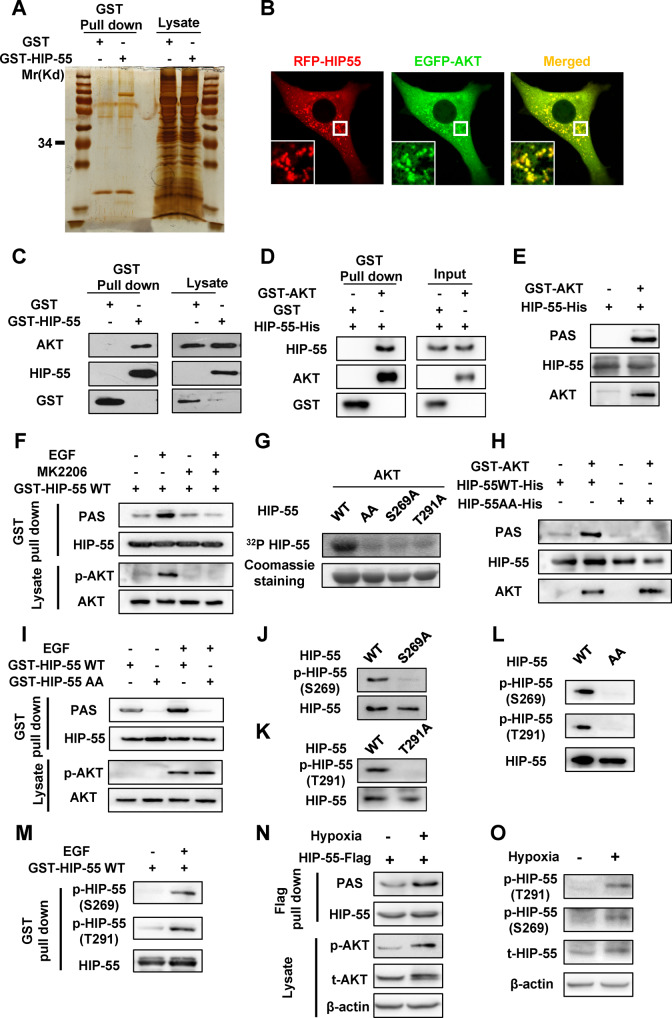
HIP-55 is a new substrate of AKT kinase. **A** Interaction proteomic screen of HIP-55. HEK293A cells were transfected with plasmids as indicated. GST-HIP-55, and its interacting proteins were enriched by GST pull-down and visualized using silver staining. HIP-55-interacting proteins were then identified by mass spectrometry. **B** Co-localization between HIP-55 and AKT. NIH-3T3 cells were co-transfected with indicated plasmids for confocal photography. *n* = 6. **C** GST pull-down confirmed HIP-55 interaction with AKT. HEK293A cells were transfected with GST or GST-HIP-55. Cell lysates were enriched with Glutathione-Sepharose 4B beads and subjected to immunoblotting. *n* = 6. **D** HIP-55 directly interacted with AKT. Purified recombinant GST or GST-AKT was incubated with purified recombinant HIP-55-His, and the interaction between AKT and HIP-55 was detected by GST pull-down. **E** HIP-55 was phosphorylated by AKT kinase. Purified recombinant HIP-55 was incubated with active AKT kinase in an in vitro kinase assay. Then the phosphorylated HIP-55 was detected by specific antibodies recognizing phospho-AKT substrates (PAS). **F** Cells were transfected with the indicated vectors and treated with 1 μM MK-2206 or vehicle (DMSO) for 1 h before stimulation with epidermal growth factor (EGF, 100 ng/ml for 10 min). HIP-55 was then enriched by GST pull-down. Phosphorylation of HIP-55 was detected using PAS antibody. *n* = 3. **G** AKT phosphorylated S269 and T291 residues of HIP-55. Purified recombinant HIP-55, HIP-55 S269A, HIP-55-T291A, HIP-55 S269A/T291A (HIP-55AA) was incubated with active AKT kinase in an in vitro kinase assay containing ^32^P-labeled ATP. Phosphorylated HIP-55 was detected by radiography. **H** Indicated purified recombinant HIP-55 was incubated with active AKT kinase in an in vitro kinase assay, and the phosphorylated HIP-55 was detected by PAS antibody. **I** AKT phosphorylated HIP-55 at S269/T291 sites in vivo. Cells were transfected with GST-HIP-55WT or GST-HIP-55AA as indicated and treated with EGF (100 ng/ml for 10 min). GST-HIP-55 was enriched by GST pull-down and then detected by PAS antibody. *n* = 3. **J** Specificity of p-HIP-55 (S269) antibody. Cells were transfected with GST-HIP-55WT or GST-HIP-55 S269A as indicated. HIP-55 was then enriched by GST pull-down and detected by the p-HIP-55 (S269) antibody. **K** Specificity of p-HIP-55 (T291) antibody. Cells were transfected with GST-HIP-55WT or GST-HIP-55-T291A as indicated. HIP-55 was then enriched by GST pull-down and detected by the p-HIP-55 (T291) antibody. **L** GST-HIP-55WT or GST-HIP-55AA was enriched by GST pull-down and then detected by the phospho-S269 and phospho-T291 of HIP-55 antibodies, respectively. **M** Cells were transfected with plasmids as indicated and then treated with EGF (100 ng/ml for 10 min). The phosphorylation of endogenous HIP-55 was detected by the p-HIP-55 (S269) and p-HIP-55 (T291) antibodies. *n* = 3. **N** Isolated cardiomyocytes were infected with the indicated adenovirus vectors and then subjected to hypoxia injury. HIP-55 was then enriched by Flag pull-down. Phosphorylation of HIP-55 was detected using PAS antibody. *n* = 4. **O** Isolated cardiomyocytes were subjected to hypoxia injury, and the phosphorylation of endogenous HIP-55 was detected by the p-HIP-55 (S269) and p-HIP-55 (T291) antibodies. *n* = 3.

To further investigate how HIP-55 controls the MAP4K1 cell death pathway, the protein interactome for HIP-55 was investigated (Fig. 6A). Notably, AKT, the most important kinase for cell survival, was found in the interactome. The interaction between HIP-55 and AKT was confirmed by confocal co-localization and GST pull-down analysis in vivo (Fig. 6B, C). The direct interaction between recombinant purified HIP-55 and AKT was also determined by GST pull-down in vitro (Fig. 6D). Furthermore, the in vitro kinase assay revealed that AKT phosphorylated HIP-55 directly, which was detected by the phospho-specific AKT substrate (PAS) antibody (Fig. 6E) and ^32^P-labeled ATP kinase assay (Fig. 6G). To further determine whether endogenous AKT phosphorylated HIP-55, epidermal growth factor (EGF) was used to activate endogenous AKT. EGF dramatically induced phosphorylation of HIP-55, which was abolished by the specific AKT inhibitor MK-2206 (Fig. 6F), indicating that endogenous AKT phosphorylated HIP-55 in vivo. Furthermore, we determined that AKT phosphorylated HIP-55 at S269 and T291. The in vitro ^32^P-labeled ATP kinase assay also showed that AKT directly phosphorylated WT HIP-55 (HIP-55WT), while S269A/T291A-mutated HIP-55 (HIP-55AA) failed to be phosphorylated by AKT (Fig. 6G). Consistently, AKT kinase phosphorylated HIP-55WT but not HIP-55AA in vitro (Fig. 6H). In vivo, HIP-55AA failed to be phosphorylated by EGF stimulation (Fig. 6I). Moreover, we generated site-specific phospho-antibodies that either recognized phospho-S269 or phospho-T291 of HIP-55. As expected, both phospho-antibodies recognized WT HIP-55, but failed to recognize either HIP-55 S269A (Fig. 6J) or HIP-55 T291A respectively (Fig. 6K), as well as HIP-55AA (Fig. 6L). Utilizing these specific antibodies, we further confirmed HIP-55 is a physiological substrate of AKT that phosphorylates at the S269 and T291 residues in vivo (Fig. 6M). Furthermore, we also observed that phosphorylation of HIP-55 S269/T291 was remarkably increased under hypoxia-induced cardiomyocyte injury by using PAS antibody (Fig. 6N) and specific phospho-antibodies of HIP-55 S269 and T291 residues (Fig. 6O).

### 14-3-3τ and HIP-55 coordinately inhibit the MAP4K1/JNK/GPX4 ferroptosis pathway in an AKT-dependent manner

**Fig. 7:**
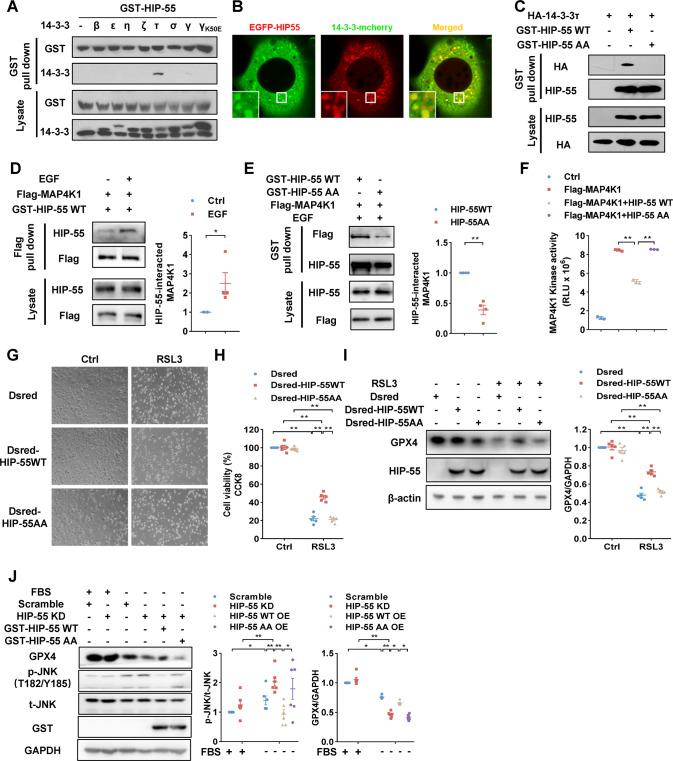
14-3-3τ and HIP-55 coordinately inhibit the MAP4K1/JNK/GPX4 ferroptosis pathway in an AKT-dependent manner. **A** Interaction assay between HIP-55 and seven isoforms of 14-3-3 proteins. HEK293A cells were co-transfected with the indicated 14-3-3 isoforms and GST-HIP-55. Interaction of HIP-55 with subtypes of 14-3-3 proteins was examined by GST pull-down. **B** Co-localization between HIP-55 and 14-3-3τ. NIH-3T3 cells were co-transfected with indicated plasmids for confocal photography. *n* = 6. **C** Phosphorylation of HIP-55 S269 and T291 were required for 14-3-3τ binding. HEK293A cells were co-transfected with the indicated vectors. GST pull-down was performed to detect interactions between 14-3-3τ with HIP-55WT or HIP-55AA. HIP-55AA mutation completely abolished the interaction between HIP-55 and 14-3-3τ. *n* = 3. **D** HIP-55 interacted with MAP4K1 in a S269/T291 phosphorylated-dependent manner. Cells were transfected with the indicated vectors and stimulated with EGF 100 ng/ml for 10 min. The interaction between HIP-55 and MAP4K1 was determined by Flag pull-down. *n* = 4. **E** Cells were transfected with the indicated vectors. Then the interaction between MAP4K1 and HIP-55WT or HIP-55AA was determined by GST pull-down. *n* = 4. **F** HIP-55 suppressed kinase activity of MAP4K1 in a S269/T291-dependent manner. Flag-MAP4K1 was co-transfected with GST-HIP-55WT or GST-HIP-55AA. Activity of MAP4K1 purified with anti-FLAG beads was detected by using ADP-Glo™ Kinase Assay. *n* = 3. **G** Cardiomyocytes were transfected as indicated and then treated with RSL3 (5 μM) for 24 h, and then imaged (magnification, ×10). *n* = 5. **H** Cell viability was determined by CCK-8 kit. *n* = 5. **I** Cardiomyocytes were transfected as indicated and then treated with RSL3 (5 μM) for 16 h, The expression of GPX4 was detected by immunoblot. *n* = 5. **J** HIP-55 inhibited JNK/GPX4 ferroptosis pathway in an S269/T291 phosphorylated-dependent manner. Cells were transfected with plasmids as indicated and then subjected to starvation for 24 h. Then the expression of GPX4 and phosphorylation of JNK were detected by immunoblot. *n* = 6. *P < 0.05, **P < 0.01. All error bars represent mean ± SEM.

AKT substrates often form a complex with 14-3-3 and function. 14-3-3 proteins are an important family of phosphoserine/phosphothreonine-binding proteins that promote cell survival. There are seven distinct 14-3-3 isoforms (β, ε, ζ, η, τ, γ and σ), which are encoded by separate genes in mammals. To examine whether HIP-55 also binds 14-3-3, we conducted a pull-down assay with HIP-55 and various 14-3-3 isoforms. HIP-55 exerted preferent interaction with 14-3-3τ, and failed to interact with the negative control 14-3-3γ/K50E mutant (Fig. 7A). Confocal co-localization analysis also demonstrated an interaction between HIP-55 and 14-3-3τ (Fig. 7B). Moreover, our research showed that the HIP-55AA mutation completely abolished the interaction between HIP-55 and 14-3-3τ (Fig. 7C), suggesting that 14-3-3τ binds HIP-55 at the AKT phosphorylation sites (HIP-55 S269 and T291) and controlled by AKT kinase. Further study showed that S269/T291 phosphorylation of HIP-55 increased the interaction between HIP-55 and MAP4K1 (Fig. 7D), whereas the HIP-55AA mutant significantly decreased this interaction (Fig. 7E). Consistently, compared with the HIP-55WT, HIP-55AA did not inhibit kinase activity of MAP4K1 (Fig. 7F). Overexpression of HIP-55WT, but not HIP-55AA, inhibited RSL3-induced cardiomyocyte ferroptosis (Fig. 7G–I). Meanwhile, overexpression of HIP-55WT, but not HIP-55AA, increased JNK/GPX4 ferroptosis pathway activation (Fig. 7J). Taken together, these results showed that AKT kinase controls the formation of the HIP-55/14-3-3τ complex and inhibits the MAP4K1-dependent JNK/GPX4 ferroptosis pathway.

### The HIP-55 signalosome-dependent ferroptosis pathway reduces myocardial infarction injury

**Fig. 8:**
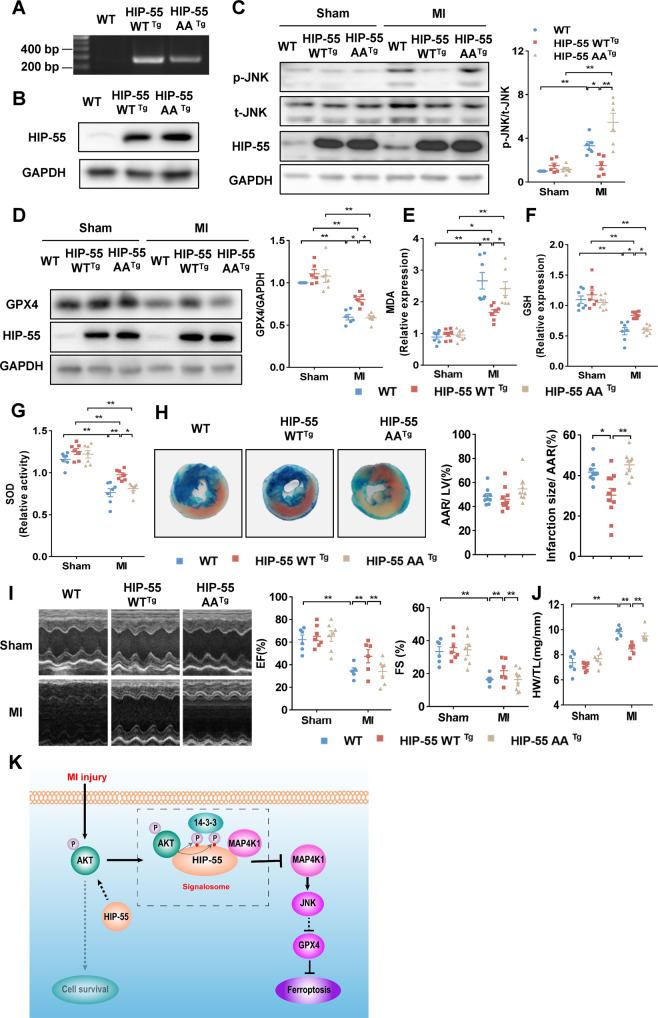
The HIP-55 signalosome-dependent ferroptosis pathway reduces myocardial infarction injury. **A** Genotyping of wildtype, HIP-55WT^Tg^ and HIP-55AA^Tg^ mice. **B** Western blots analysis of cardiac proteins from WT, HIP-55WT^Tg^, and HIP-55AA^Tg^ mice displaying overexpression of HIP-55 in the transgenic hearts. **C** HIP-55 inhibited MI-induced JNK activation in a S269/T291-dependent manner. Western blots analysis of JNK phosphorylation in the heart tissues of WT, HIP-55WT^Tg^ and HIP-55AA^Tg^ mice after MI. *n* = 5. **D** HIP-55 inhibited MI-induced cardiomyocyte ferroptosis relies on its S269/T291 phosphorylation state. Representative immunoblots and statistical data of GPX4 expression in heart tissue from WT, HIP-55WT^Tg^, and HIP-55AA^Tg^ mice after MI. *n* = 6. **E** Relative expression of MDA in heart tissue from WT, HIP-55WT^Tg^ and HIP-55AA^Tg^ mice after MI. *n* = 7. **F** Relative expression of GSH in heart tissue from WT, HIP-55WT^Tg^ and HIP-55AA^Tg^ mice after MI. *n* = 7. **G** Relative activity of SOD in heart tissue from WT, HIP-55WT^Tg^ and HIP-55AA^Tg^ mice after MI. *n* = 7. **H** HIP-55 suppressed myocardial infarction size depends on its S269/T291 phosphorylation. Representative Alcian blue-TTC staining and statistical data for area at risk and infarct size in heart tissue from WT, HIP-55WT^Tg^, and HIP-55AA^Tg^ mice post-MI. WT, *n* = 9; HIP-55WT^Tg^, *n* = 11; HIP-55AA^Tg^, *n* = 9. **I** HIP-55 suppressed MI-induced cardiac contractile dysfunction in an S269/T291-dependent manner. Representative M-mode echocardiographic photographs (left) and cardiac contractile function (right) quantified by echocardiographic analysis of ejection fraction (EF) and fractional shortening (FS) in WT, HIP-55WT^Tg^, and HIP-55AA^Tg^ mice after MI. *n* = 5–7. **J** HIP-55 alleviated cardiac hypertrophy after MI depends on its S269/T291 phosphorylation. The ratio of heart weight to tibial length (HW/TL) in WT, HIP-55WT^Tg^ and HIP-55AA^Tg^ mice. *n* = 6–7. *P < 0.05, **P < 0.01. All error bars represent mean ± SEM. **K** The schematic diagram of HIP-55-dependent signalosome determines cardiomyocyte fate. HIP-55 functions as the central hub to assemble AKT, 14-3-3τ and MAP4K1 to form a signalosome, where AKT phosphorylates HIP-55 at its S269/T291 sites and HIP-55 then rewires AKT signaling to negatively regulate the MAP4K1 cell death pathway against ferroptosis and MI injury in a site-specific manner.

The molecular mechanism described above indicates that the HIP-55 signalosome, including AKT, 14-3-3 and MAP4K1, functions as a critical determinant in the fate of cardiomyocyte fate at the cellular level. To provide further evidence in the animal MI model, we developed cardiac-specific overexpression of HIP-55AA transgenic mice (HIP-55AA^Tg^) (Fig. 8A, B). Indeed, MI-induced activation of JNK was obviously suppressed in cardiac-specific overexpression of HIP-55WT^Tg^ mice, but not in HIP-55AA^Tg^ mice (Fig. 8C). Furthermore, for cardiac-specific overexpression of HIP-55WT^Tg^ mice, but not HIP-55AA^Tg^ mice, the expression of GPX4 was markedly increased, and lipid peroxidation and oxidative stress was alleviated after MI (Fig. 8D–G). Importantly, the infarction size was significantly reduced in cardiac-specific overexpression of HIP-55WT^Tg^ mice, but not HIP-55AA^Tg^ mice (Fig. 8H). Consistently, the HIP-55WT^Tg^ mice, but not HIP-55AA^Tg^ mice, showed significant improvement in cardiac contractile function and inhibition of cardiac hypertrophy after MI (Fig. 8I, J). Taken together, these results indicated that the HIP-55-mediated signalosome was required to protect cardiomyocytes against ferroptosis and MI injury in the animal model.

In summary, HIP-55 is a new substrate of AKT kinase and, is phosphorylated by AKT at S269/T291. The phosphorylated HIP-55 recruits 14-3-3τ to form a HIP-55/14-3-3τ complex that then inhibits the MAP4K1/JNK/GPX4 cell death pathway, leading to cardiac protection after MI (Fig. 8K). Thus, HIP-55 functions as the hub of a signalosome contributing to endogenous protection against MI-induced ferroptosis and injury.

## Discussion

Although ferroptosis is strongly implicated in MI, the complicated regulation mechanisms of ferroptosis, especially the cross-talk with classical survival/death signals, remain poorly understood. This study reveals that the adaptor protein HIP-55 integrates and coordinates the classical AKT survival/MAPK death pathways and ferroptosis in MI.

AKT is a classical and general mediator of cell survival and has been shown to suppress death in a number of cell types [12]. In the heart, constrained by the limited proliferative capacity of cardiomyocytes, the AKT pathway plays a key role in regulating cardiomyocyte survival, with little effect on proliferation [13]. Many cellular pro-survival factors, such as IGF-1, FGF, and others, have also been shown to protect myocytes from MI injury and to be dependent on AKT-mediated cell survival signaling [14, 15]. AKT inhibition of myocyte death can be induced by various stimuli, including serum deprivation, MI, ischemia-reperfusion, and cell-cycle disruption, suggesting that AKT lies at the crossroads of multiple apoptotic and nonapoptotic signals activated during myocardial injury [12]. Notably, some studies have demonstrated that the activation of the AKT signaling pathway also suppresses ferroptosis [16, 17], and impaired AKT signaling triggers disturbances in iron metabolism [18].

It is well known that the central component of the regulation of cell death is a member of the MAP kinase family, especially JNK MAPK and p38 MAPK [19]. Recent studies have shown that the JNK and p38, but not ERK pathways mediated erastin-induced ferroptosis in human promyelocytic leukemia [20, 21]. However, studies have also indicated both protective and detrimental roles for p38 MAPK in myocardial injury. For example, p38 MAPK activation in mice contributes to acute myocardial injury and death, while the same activation in pig has no causative effects [22]. In addition, p38 kinase rescues failing myocardium through angiogenic and anti-cell death processes after MI [23]. These results suggest that the role of p38 MAPK in myocardial injury is still controversial. There is, however, abundant evidence that activation of JNK MAPK is critical for induction of cell death after MI [24–27]. JNK MAPK has been shown to be involved in a wide array of cell death signaling. Through its interaction with a diverse set of signaling proteins and adaptors, JNK have been shown to play a key role in apoptotic and nonapoptotic programmed cell death mechanisms including those of pyroptosis, necroptosis, ferroptosis, and autophagy [11]. Most of the functions of JNK MAPK can be related to its ability to modulate cell death via these programmed cell death mechanisms. Iron overload can activate JNK MAPK and impair insulin sensitivity in human skeletal muscle cells, suggesting that the JNK MAPK pathway may be involved in ferroptosis [26]. Further studies demonstrated that Fe^2+^ and I/R-stress induced JNK activation and translocation to the mitochondria and increased I/R-induced lipid peroxidation, infarct size and cardiomyocyte death [27, 28].

Although the above evidence clearly shows that AKT and MAPK are involved in the regulation of ferroptosis, how the most important and classical survival/death (AKT/MAPK) pathways are integrated into the ferroptosis pathway, a novel type of cell death, is still a burning issue. AKT functions as the most important core node of the cell survival signaling network via its diverse substrates. Currently, more than 20 AKT substrates have been rigorously identified, including endothelial nitric oxide synthase, vascular endothelial growth factor, mammalian target of rapamycin, glycogen synthase kinase 3β, and forkhead box subfamily O, among others [12]. These substrates could be divided into different functional classes, such as transcription factors, cell-cycle regulators, or kinases [12]. The diversity of downstream substrates may be utilized by AKT to discriminate and differentially respond to a wide variety of extracellular stimuli. Here we identified a new type of AKT substrate: an adaptor protein HIP-55. HIP-55 provides an architectural platform for molecular assembly and may integrate AKT into many distinct pathways. It is well known that the central components of the regulation of cell death are members of the MAP kinase family. In this study, we found AKT signaling was rewired by HIP-55 to control the MAP4K1/JNK cell death pathway. MAP4K1 is a hematopoietic‐specific mammalian STE20‐like protein serine/threonine kinase and is crucial to MAPK signaling cascades and cell death [10]. MAP4K1 can specifically activate the JNK pathway, but does not stimulate p38 signaling pathways [10]. Thus, in an AKT/HIP-55/MAP4K1 signalosome, AKT can precisely regulate a JNK-mediated cardiomyocyte death pathway, but not a p38 pathway after MI.

14-3-3τ is another vital component of the HIP-55-dependent signalosome. Several studies have concluded that 14-3-3 proteins are involved in cardiomyocyte survival [29–31]. 14-3-3 proteins function by binding to various client proteins. Dominant negative 14-3-3 transgenic cardiac tissue had a profound increase in cardiomyocyte mortality after transverse aortic constriction [32]. There are seven mammalian members of the 14-3-3 family encoded by separate genes (β, γ, ϵ, η, σ, τ and ζ) [32]. Using a pull-down assay, HIP-55 was found to preferentially interact with the 14-3-3τ isoform. Excitingly, a previous study has shown that the 14-3-3τ phosphoserine-binding protein is required for cardiomyocyte survival [33]. 14-3-3τ^+/−^ mice had increased LV chamber dilation and increased infarct size. Furthermore, 14-3-3τ^+/−^ mice had significantly increased mortality after MI. Those evidence strongly support our results that HIP-55 can be phosphorylated by AKT and recruit 14-3-3τ to inhibit the MAP4K1 cell death pathway and protect cardiomyocytes against MI-induced ferroptosis injury.

Of course, there are still some limitations in the present study. First, the present conclusions are based on findings derived from animal models. The correlation between the findings and human MI needs to be further studied. Especially, it is key point to determine the contribution of the mutation sites (S269/T291) on HIP-55 for human MI. Second, the present research lacks the exploration of translational medicine. The development of small molecule drugs or other specific methods to activate AKT/HIP-55 pathway will be a valuable research strategy for the treatment of human MI.

In summary, as a recently identified form of cell death, ferroptosis has been identified as a key contributor to MI-induced cardiomyocyte death. Understanding the relationship between ferroptosis mechanism and classical survival/death signal may provide new diagnostic and therapeutic approaches to regulate cell survival and death in human diseases.

## Materials and methods

### Animals

All animal studies were approved by the Biomedical Research Ethics Committee of Peking University and were compliant with the National Institutes of Health Guide for the Care and Use of Laboratory Animals. The whole-body HIP-55-knockout mice (HIP-55^−/−^, C57BL/6J background) were generated using TALEN technology by Cyagen Biosciences Inc. (Guangzhou, Guangdong, China). Cardiac-specific overexpression of HIP-55 mice (HIP-55^Tg^, C57BL/6J background) was constructed by Cyagen Biosciences Inc. Genomic DNA was extracted from tail biopsies for genotyping. The primers for HIP-55 knockout mice were: forward, 5′-AAGTGCTGGGATTAAAGGCGTGC-3′; reverse, 5′-GTCACTGTAGCTGAGCTGGAGGAAGA-3′. The primers for cardiac-specific overexpression of HIP-55WT mice (HIP-55WT^Tg^) were: forward, 5′-GATCTCCTTTGACCCCGAGAACCTCA-3′; reverse, 5′-CTGGGTTTACTTGTCATCGTCGTCCT-3′. The primers for cardiac-specific overexpression of HIP-55 S269A/T291A mice (HIP-55AA^Tg^) were: forward, 5′-ATGACAGACAGATCCCTCCTATCTCC-3′; reverse, 5′-GCCTTCATAGGTAAAGAGAGCCCAGTCG-3′.

### Isolation of cardiomyocytes

Neonatal rat ventricular cardiomyocytes (NRVMs) were isolated from neonatal Sprague-Dawley rats as described previously [34]. NRVMs were then cultured in Dulbecco’s Modified Eagle Medium with 10% fetal bovine serum and antibiotics (50 mg/ml streptomycin, 50 U/ml penicillin) at 37 °C and 5% CO_2_.

### Hypoxia-induced injury of cardiomyocytes

The isolated cardiomyocytes were cultured under normal conditions for 48 h, then the cardiomyocytes were cultured in serum-free, low glucose (1 g/l) and hypoxic (0.1% O_2_) conditions 24 h to simulate the ischemic state of MI.

### Cell viability assay

To measure cell viability, cardiomyocytes were seeded in 96-well plates and treated as indicated. Cardiomyocyte viability was then assessed by Cell Counting Kit-8 (CCK-8, #HY-K0301, MedChemExpress) in accordance with the manufacturer’s instructions.

### Plasmids and transfection

The GST-HIP-55 plasmid was generated by inserting the human HIP-55 cDNA, along with an N-terminal GST tag, into the Gateway expression vectors (Thermo Fisher). The site mutant plasmids were generated using the QuikChange Site-Directed Mutagenesis Kit (Stratagene) according to the manufacturer’s instructions. EGFP-MAP4K1, EGFP-AKT, and EGFP-14-3-3τ plasmids were constructed by subcloning the corresponding vector DNAs into the pEGFP-N1 vector using the EasyGeno Assembly Cloning Kit (TIANGEN Biotech). The AKT vector DNAs were a gift from Prof. Chundong Yu (Xiamen University, Xiamen, China). Cells were transfected with plasmids using Lipofectamine 3000 (Invitrogen) following the manufacturer’s instructions.

### Antibodies and chemicals

Antibodies to phospho-AKT (Ser473) (1:5000, #4060), pan-AKT (1:2000, #9272), GST (1:3000, #2624), HA (1:3000, #3724), phospho-JNK(T182/Y185) (1:2000, #4668), pan-JNK (1:2000, #9252), phospho-AKT substrate (1:5000, #9614) and GAPDH (1:5000, #5174) were from Cell Signaling Technology. Antibody to HIP-55 (sc-366772, #1:2000) and 14-3-3 (1:2000, #sc-629) was from Santa Cruz Biotechnology. Antibodies to Flag (1:5000, #F3165) and Anti-Flag M2 affinity gel (#A2220) were from Sigma- Aldrich. Antibodies to GPX4 (1:3000, #ab125066) were from Abcam. Anti-phospho-HIP-55 (phosphorylated at S269) polyclonal antibodies (1:1000) were generated in rabbits against the synthesized phosphopeptide (KERAMpSTTS). Anti-phospho-HIP-55 (phosphorylated at T291) polyclonal antibodies (1:1000) were generated in rabbits against the synthesized phosphopeptide (FLQKQLpTQPE). Glutathione-Sepharose 4B was from GE Healthcare. Collagenase II (#1148090), DAPI (#D9542) and Hoechst (#94403) were from Sigma-Aldrich. RSL3 (#HY-100218A), Fer-1 (#HY-100579) and MK-2206 (#HY-10358) were from MedChemExpress.

### Myocardial infarction surgery-left anterior descending artery occlusion

Male mice were used as experiment subjects. After induction of isoflurane anesthesia, mice were placed and fixed in a right lateral position to fully expose the left chest with its paws gently taped onto the circulatory heating board. Surgery was performed using a microscope. The third intercostal space was exposed and delicately dissected until the left coronary artery (LAD) was found. The LAD was ligated with 8–0 silk sutures. The chest and skin were closed with 5–0 silk sutures. The mice in the sham group underwent the same procedure except for the ligation of the LAD.

### Echocardiography

Transthoracic two-dimensional echocardiography was performed on anaesthetized mice to evaluate cardiac function using a Vevo 2100 high-resolution microimaging system (Visualsonic Inc., Toronto, Canada). The ejection fraction and fractional shortening were calculated as previously described [35].

### Alcian blue-TTC staining

To determine infarct size, 1% Alcian blue was injected into the aorta, and the heart was rapidly excised, washed and sectioned. After removing the right ventricle, each slice was incubated at 37 °C with 1% TTC for 10 min and then fixed in 10 % formalin for 6–8 h. Image-Pro Plus 6 was used to calculate the area of the left ventricle (LV, all myocardium), the area at risk (AAR, red myocardium and white myocardium) and the size of infarct area (white myocardium) in each slice of myocardium, and a correction was made using weight ratio (%). AAR/LV was used to evaluate the consistency of the surgery, and IA/AAR was used to evaluate the area of MI.

### Immunofluorescence

For tissue immunofluorescence staining, heart tissue was fixed in 4% paraformaldehyde and dehydrate with 30% sugar and embedded in OCT for frozen section (6 μm) preparation. For cellular immunofluorescence staining, cardiomyocytes were cultured in a confocal dish. The heart tissue sections or cardiomyocytes were blocked for 1 h with PBS containing 10% goat serum and then incubated with the primary antibodies overnight. Tissue sections or cell cultures were then incubated with Alexa Fluor 488 conjugated goat anti-rabbit secondary antibody for 1 h. Subsequently, Hoechst was added and incubated for 10 min at room temperature. A Zeiss 780 laser scanning confocal microscope (Carl Zeiss Canada Ltd., Toronto, Ontario, Canada) was used to capture the immunofluorescence images.

### Detection of malondialdehyde (MDA)

Analysis of lipid peroxidation was assessed by quantification of MDA concentration in heart tissues using Lipid Peroxidation MDA Assay Kit (Jiancheng Bioengineering Institute) in accordance with the manufacturer’s instructions.

### Measurement of GSH and SOD

Analysis of redox state was assessed by quantification of GSH and SOD in heart tissues using GSH Kit (Jiancheng Bioengineering Institute) and SOD Kit (Jiancheng Bioengineering Institute) respectively.

### Confocal microscopy

For co-localization analysis, cells were transfected with the indicated plasmids for 48 h. Then the cellular microscopy was captured using the Zeiss 780 laser scanning confocal microscope.

### Pull-down assays

Cell extracts were prepared using lysis buffer (1% NP-40, 150 mM NaCl, 100 mM Hepes, 5 mM Na_4_P_2_O_7_, 5 mM NaF, 2 mM Na_3_VO_4_, 1 mM phenylmethylsulfonyl fluoride, 10 mg/l aprotinin, 10 mg/l leupeptin, PMSF).

For GST pull-down assay, the lysates were incubated with Glutathione-Sepharose 4B beads (GE Healthcare) at 4 °C 4 h on rotation. After three washes with lysis buffer, the bound proteins were released in SDS loading buffer.

For the Flag pull-down assay, the lysates were first incubated with anti-Flag M2 affinity gel (Sigma) at 4 °C overnight with rotation. After incubation, complexes were washed three times with lysis buffer, and the bead-conjugated proteins were released with SDS loading buffer.

### Protein interactome analysis of HIP-55

GST-HIP-55 plasmids were transiently transfected into cells, and proteins interacting with HIP-55 were enriched by GST pull-down assay as described above. The enriched proteins were separated by SDS-PAGE and visualized by silver staining. Protein bands were cut from the gels and digested with trypsin, and then peptides were analyzed using an LC-MS/MS platform with a nanoACQUITY ultra-performance liquid chromatography system (Waters, Milford) and an LTQ Orbitrap Velos mass spectrometer (Thermo Fisher Scientific) as described previously [36].

### In vitro kinase assay

For in vitro kinase assays, recombinant active kinases and substrates were added to 30 μl kinase buffer containing 10 μCi (γ-^32^P) ATP. Kinase reactions were performed at 30 °C for 30 min and stopped by the addition of SDS loading buffer. Reaction products were separated by SDS-PAGE and transferred to nitrocellulose membranes. Then radiolabeled proteins were visualized by autoradiography.

### In vivo kinase assay

For in vivo kinase assays, Flag-tagged kinases were enriched via Flag pull-down. Kinase activity was then detected using ADP-Glo assay (Promega, Fitchburg, WI) according to the manufacturer’s instructions. In brief, ADP-Glo is performed in two steps after kinase reaction. First, terminated the kinase reaction and eliminated the remaining ATP. Second, converted the generated ADP to ATP, and then measured the newly produced ATP using a luciferase reaction. The luminescent signal is positively correlated with kinase activity.

### Western blotting

Equal protein lysates from cells or heart tissues were separated on SDS-PAGE, then transferred onto nitrocellulose membrane. Membranes were then incubated with appropriate primary antibodies, followed by horseradish peroxidase–conjugated secondary antibodies, and detected by enhanced chemiluminescence reagents.

### Quantitative RT-PCR

Total RNA was isolated from heart tissues with TRIzol reagent and reverse transcribed by using TransScript® II Two-Step RT-PCR SuperMix System (Transgenbiotech). Quantitative RT-PCR was performed with the GoTaq qPCR Master Mix (Promega) and specific primers in the Stratagene Mx3000P qPCR System. The primers for HIP-55 (mouse) were: forward, 5′-CGCGTAGTCACCGAGAAATCC-3′; reverse, 5′-GCTCCTTTCCGCACATCATTC-3′. The primers for GAPDH (mouse) were: forward, 5′-ATGTTCCAGTATGACTCCACTCACG-3′; reverse, 5′-GAAGACACCAGTAGACTCCACGACA-3′.

### Statistical analysis

Statistics were performed using GraphPad Prism 8.0. Data are presented as mean ± SEM. Differences between two groups with normal distributions were analyzed by the two-tailed Student’s *t*-test. Comparisons between multiple groups were assessed by one-way or two-way analysis of variance with Tukey’s multiple comparisons test. In all cases, statistical significance was indicated at *P* < 0.05.

## Supplementary information

Uncropped original western blots

CDD-checklist

CDD-author-contribution-form

## Data Availability

All data are available from the corresponding author upon reasonable request. Original western blots images are available at Supplementary Materials.
