# Supplementary material for: Adaptor protein HIP-55-mediated signalosome protects against ferroptosis in myocardial infarction
Source: Cell Death Differ. 2023 Jan 13;30(3):825–38. doi: 10.1038/s41418-022-01110-z (PMC9984488; doi:10.1038/s41418-022-01110-z)

Uncropped images from Western blots

Uncropped images for Fig. 1

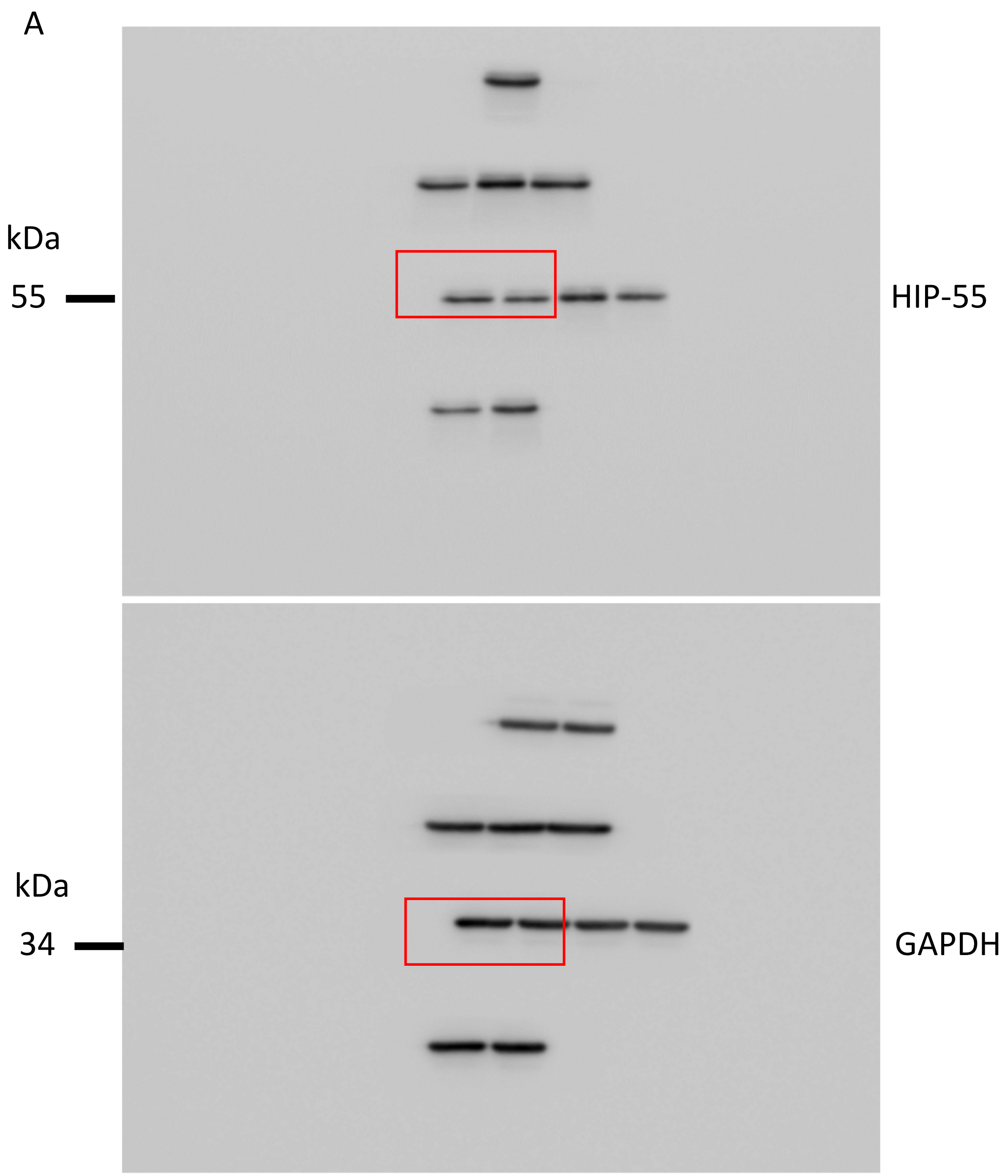

Uncropped images from Western blots

Uncropped images for Fig. 1

C

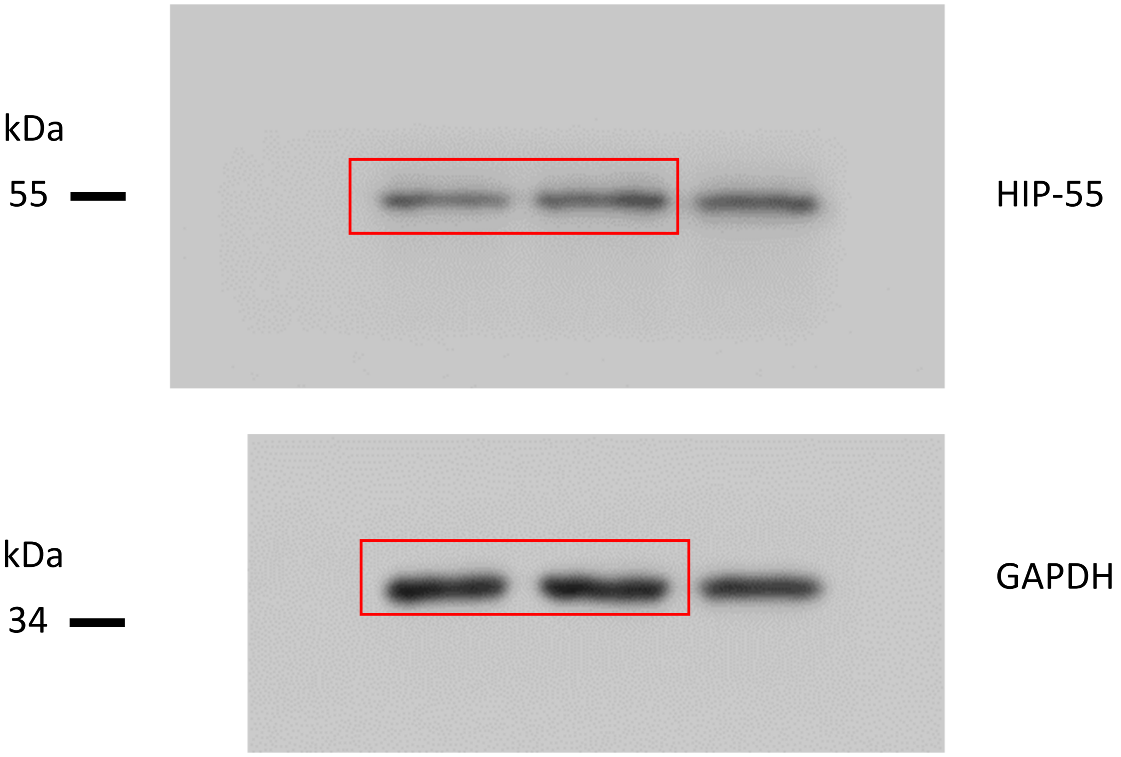

Uncropped images from Western blots

Uncropped images for Fig. 1

F

kDa

55

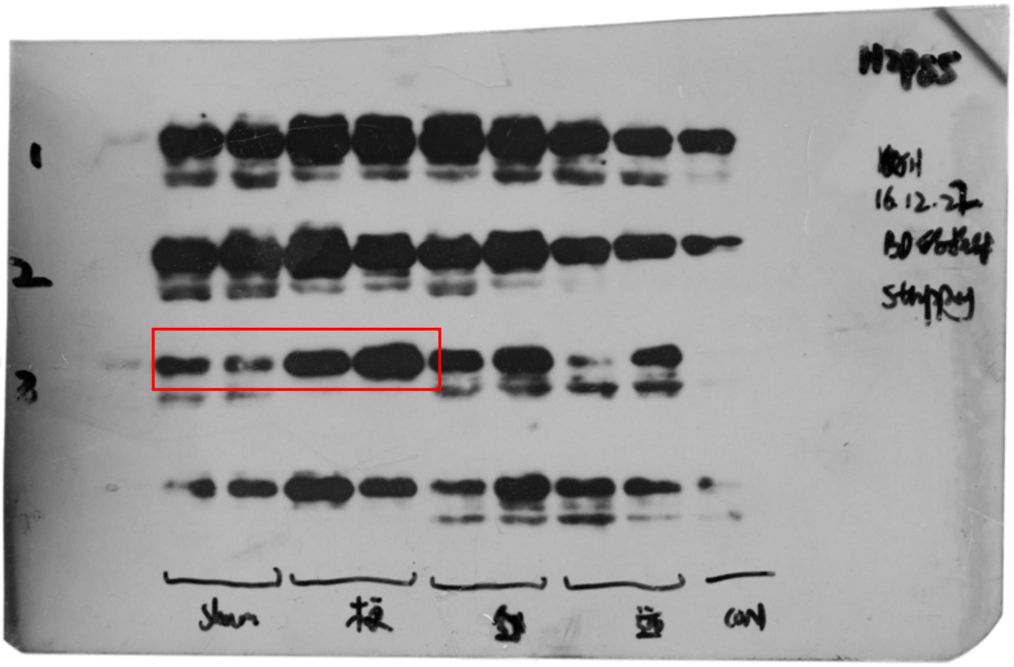

HIP-55

kDa

34

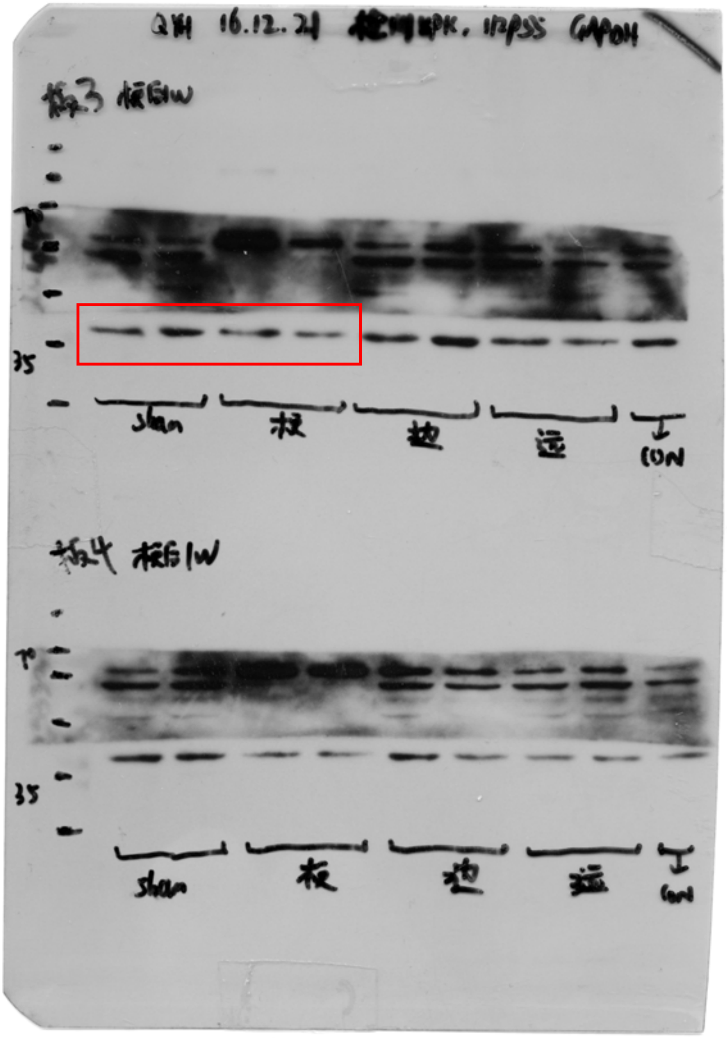

GAPDH

Uncropped images from Western blots

Uncropped images for Fig. 2

A

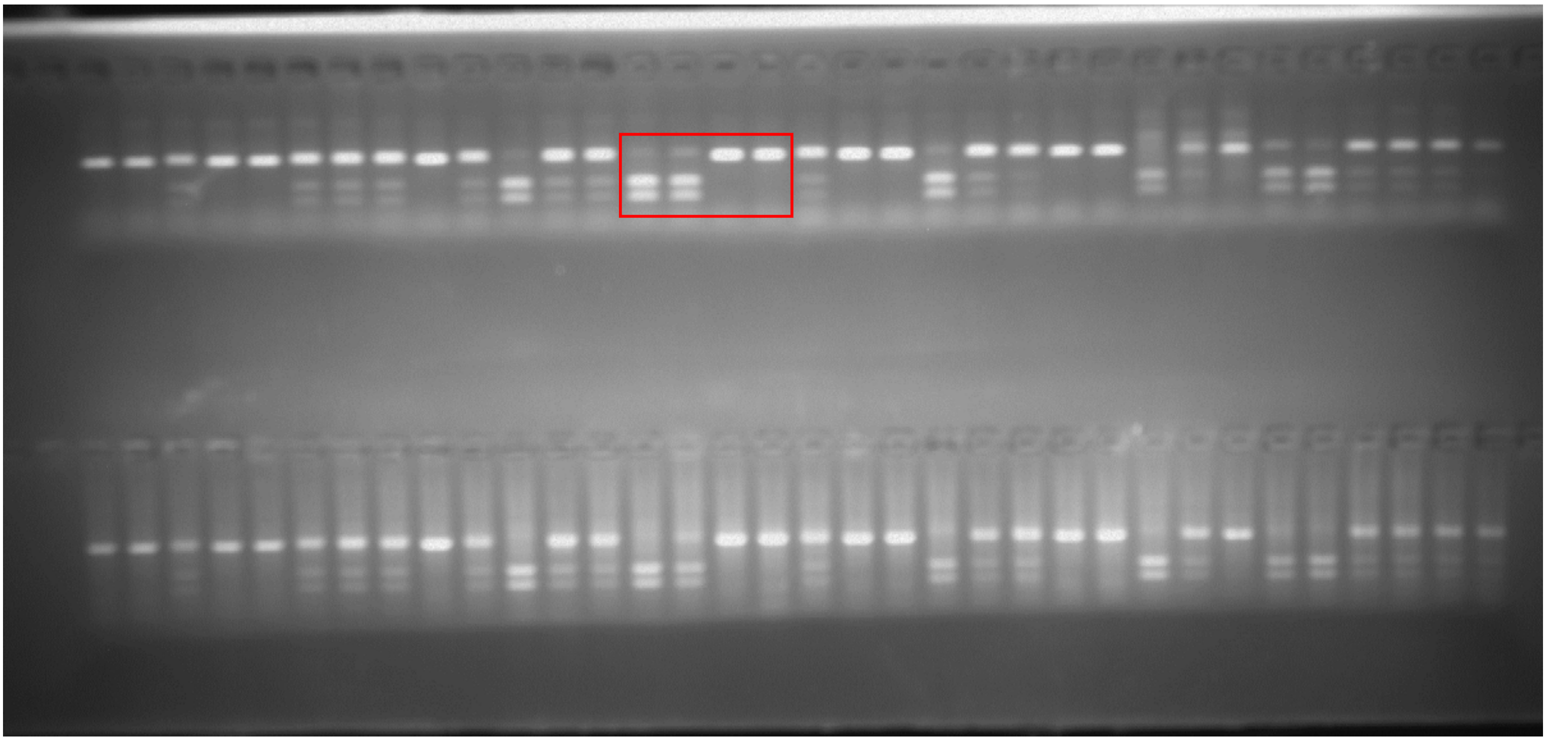

B

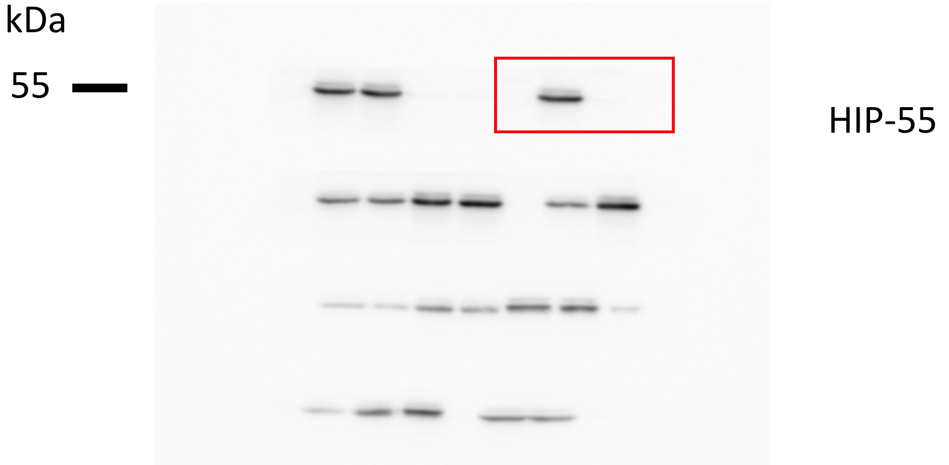

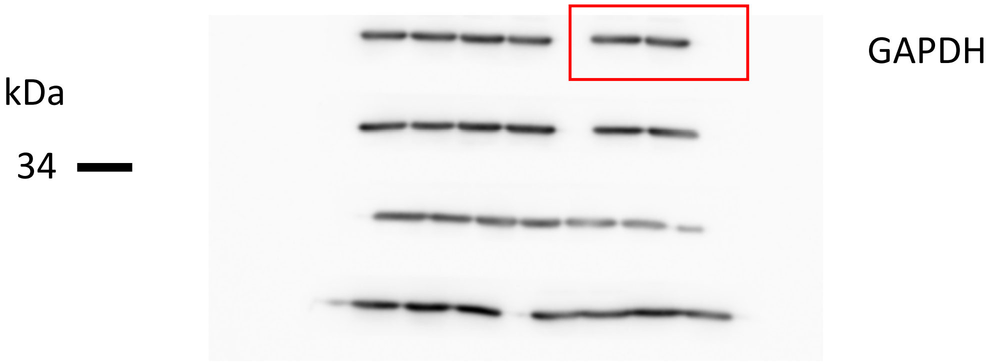

Uncropped images from Western blots

Uncropped images for Fig. 3

A

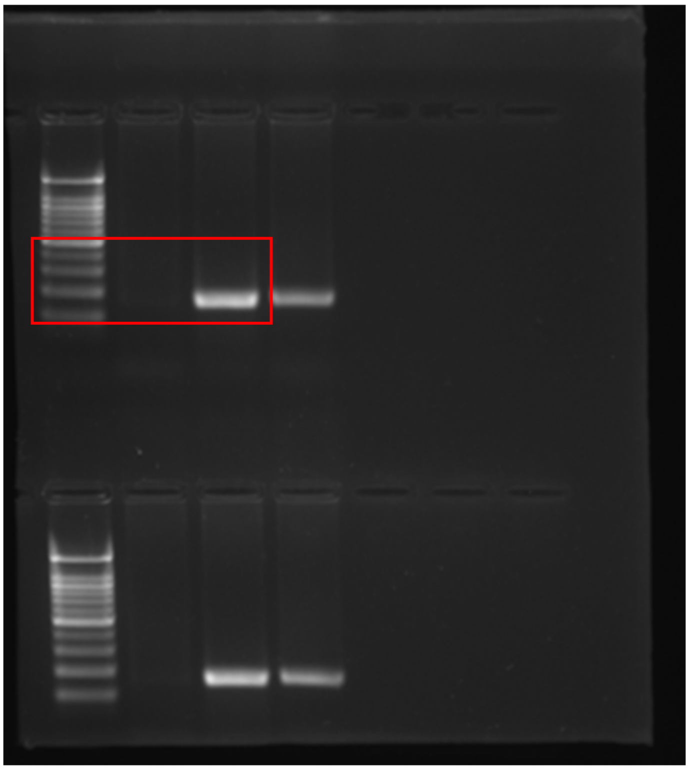

B

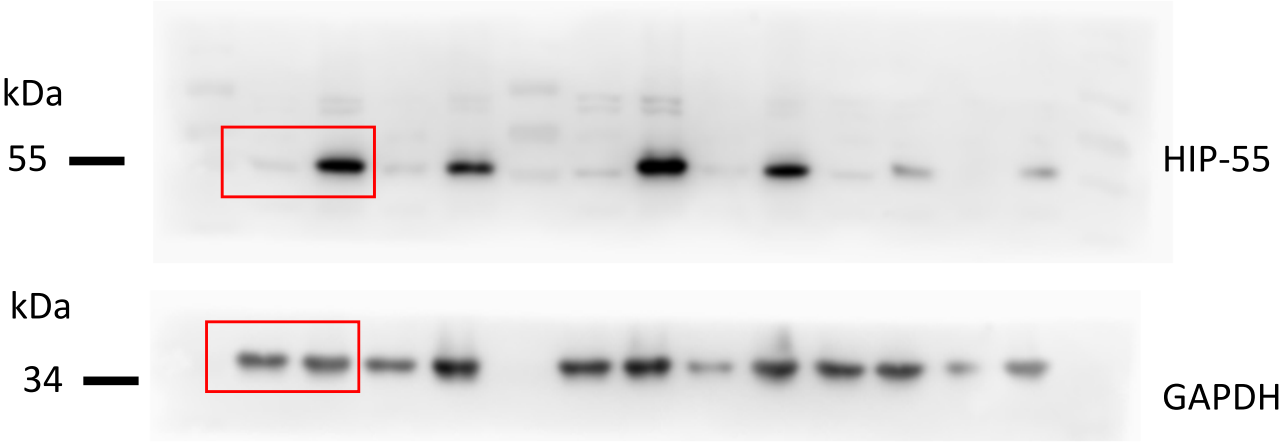

Uncropped images from Western blots

Uncropped images for Fig. 4

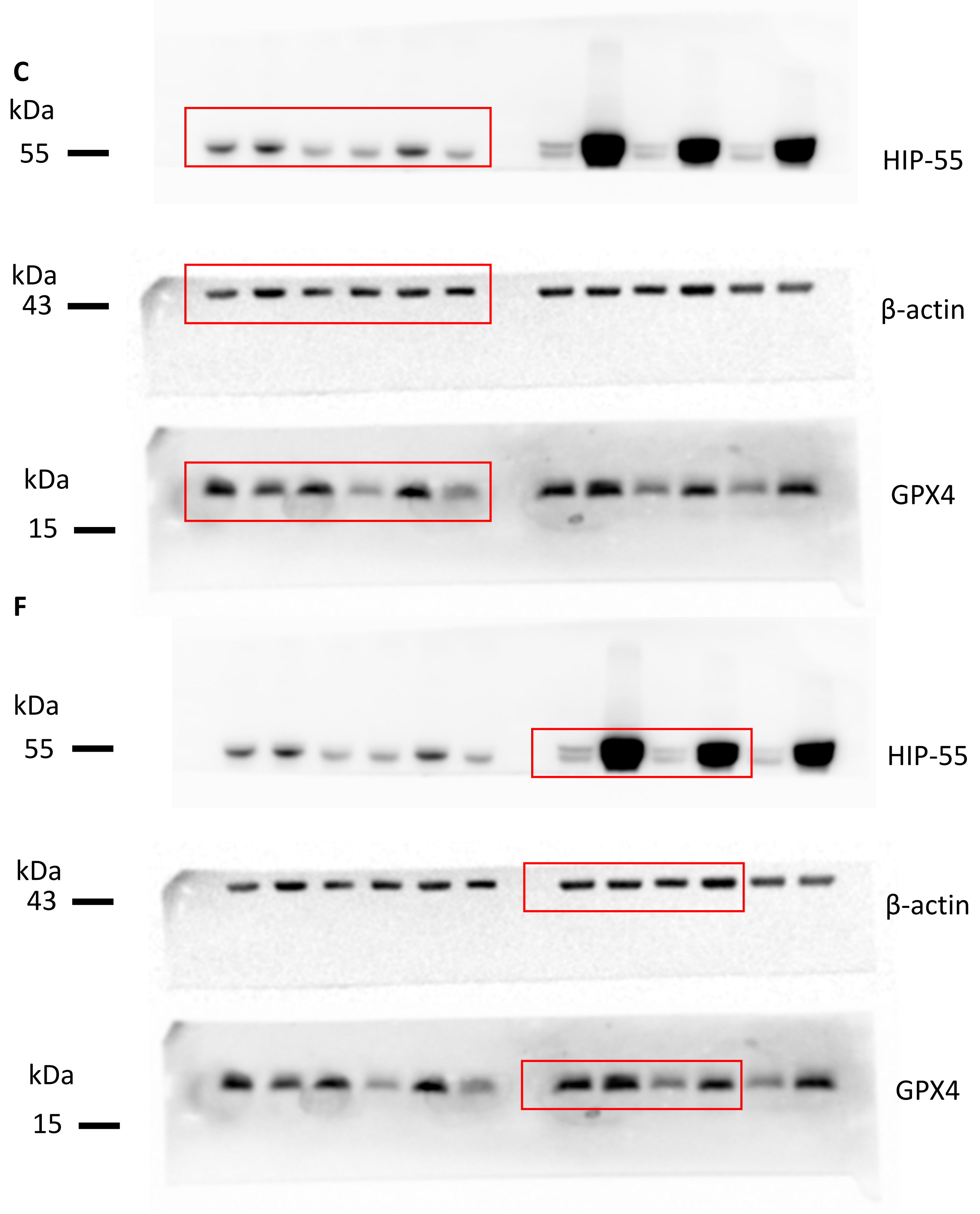

Uncropped images from Western blots

Uncropped images for Fig. 4

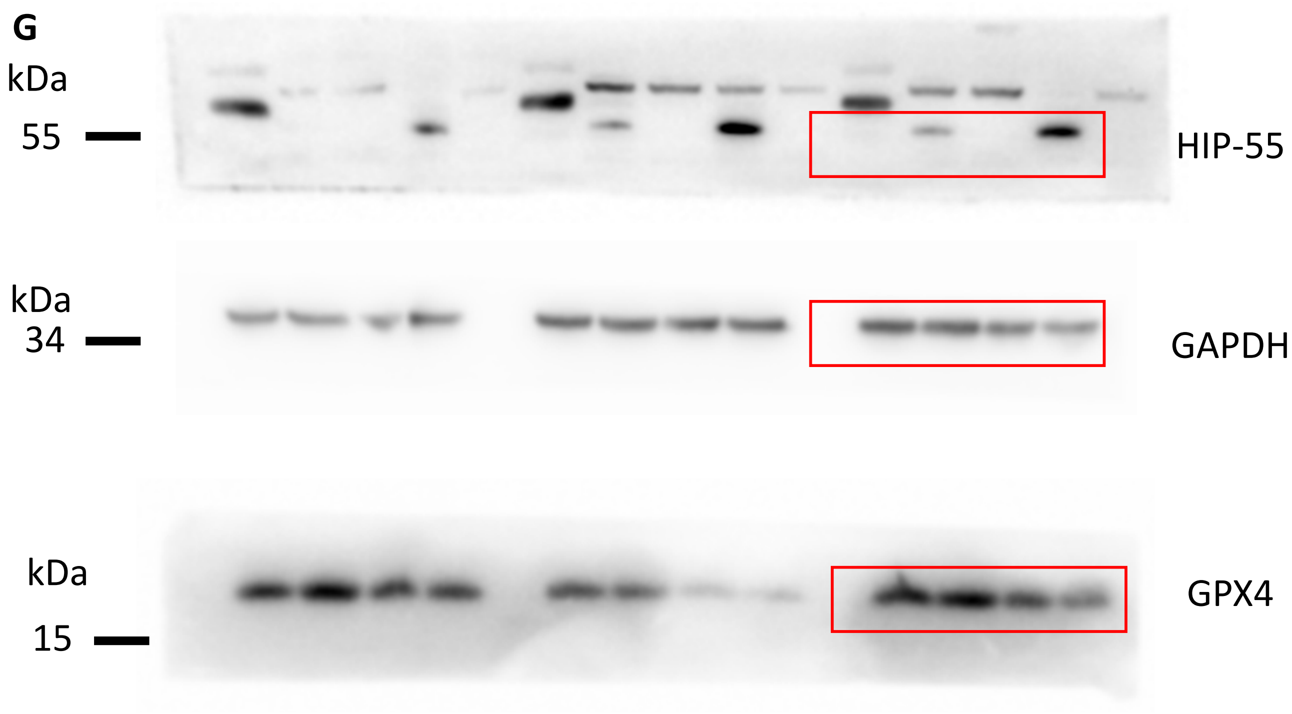

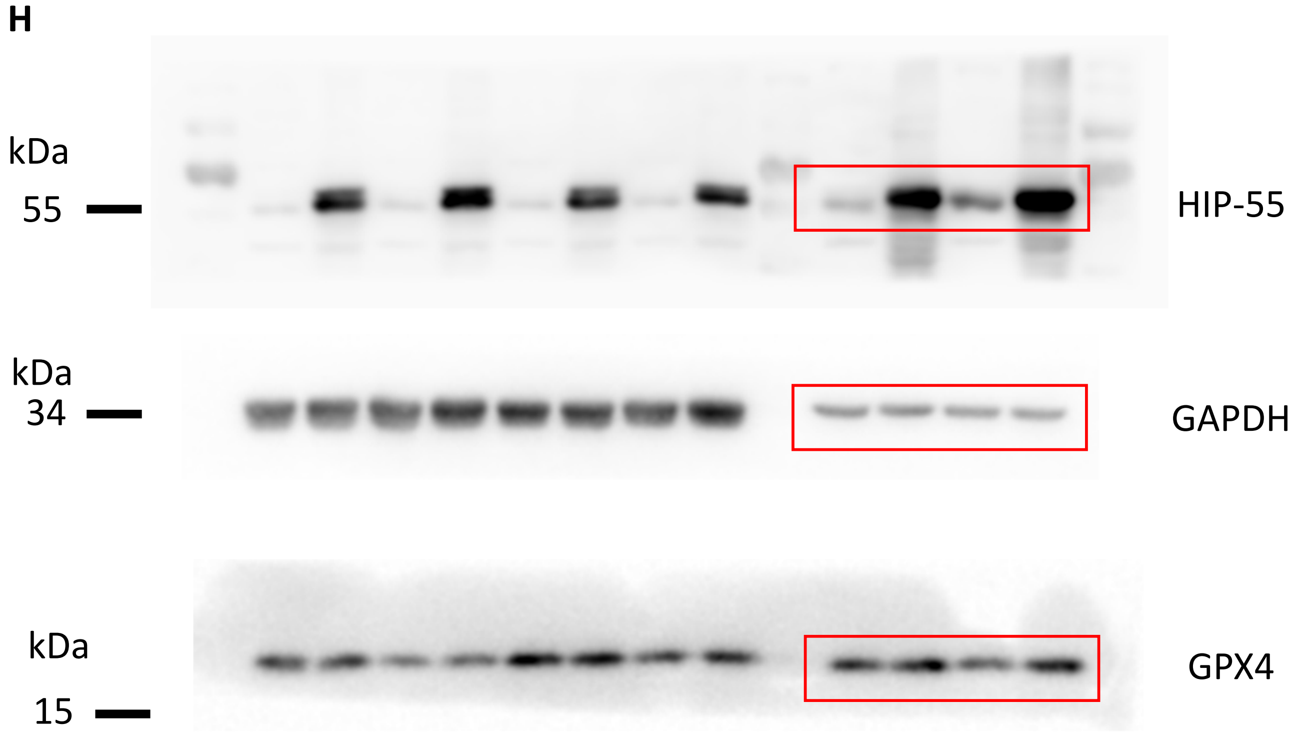

Uncropped images from Western blots

Uncropped images for Fig. 5

A **Upper panel** **Lower panel**

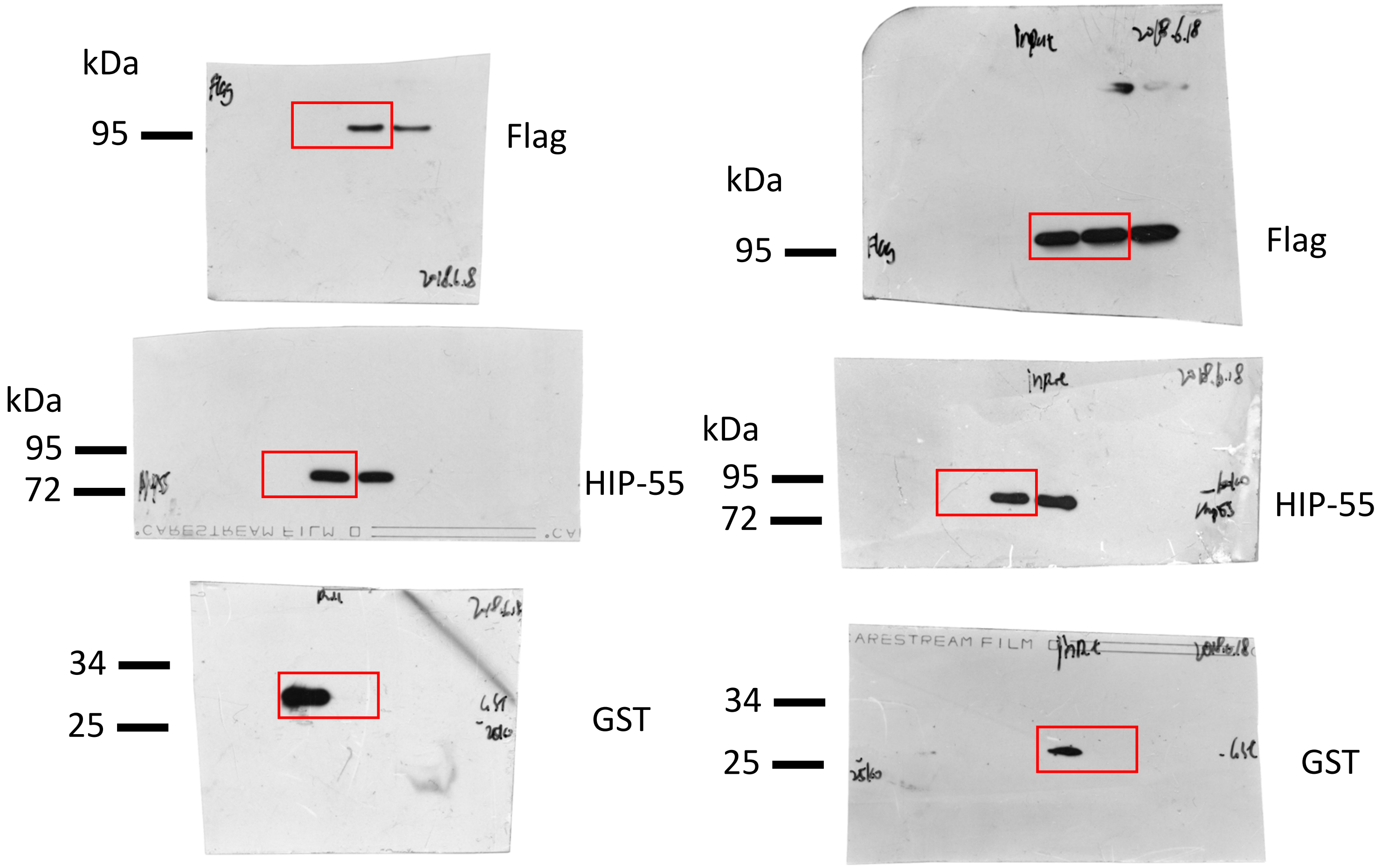

Uncropped images from Western blots

Uncropped images for Fig. 5

D

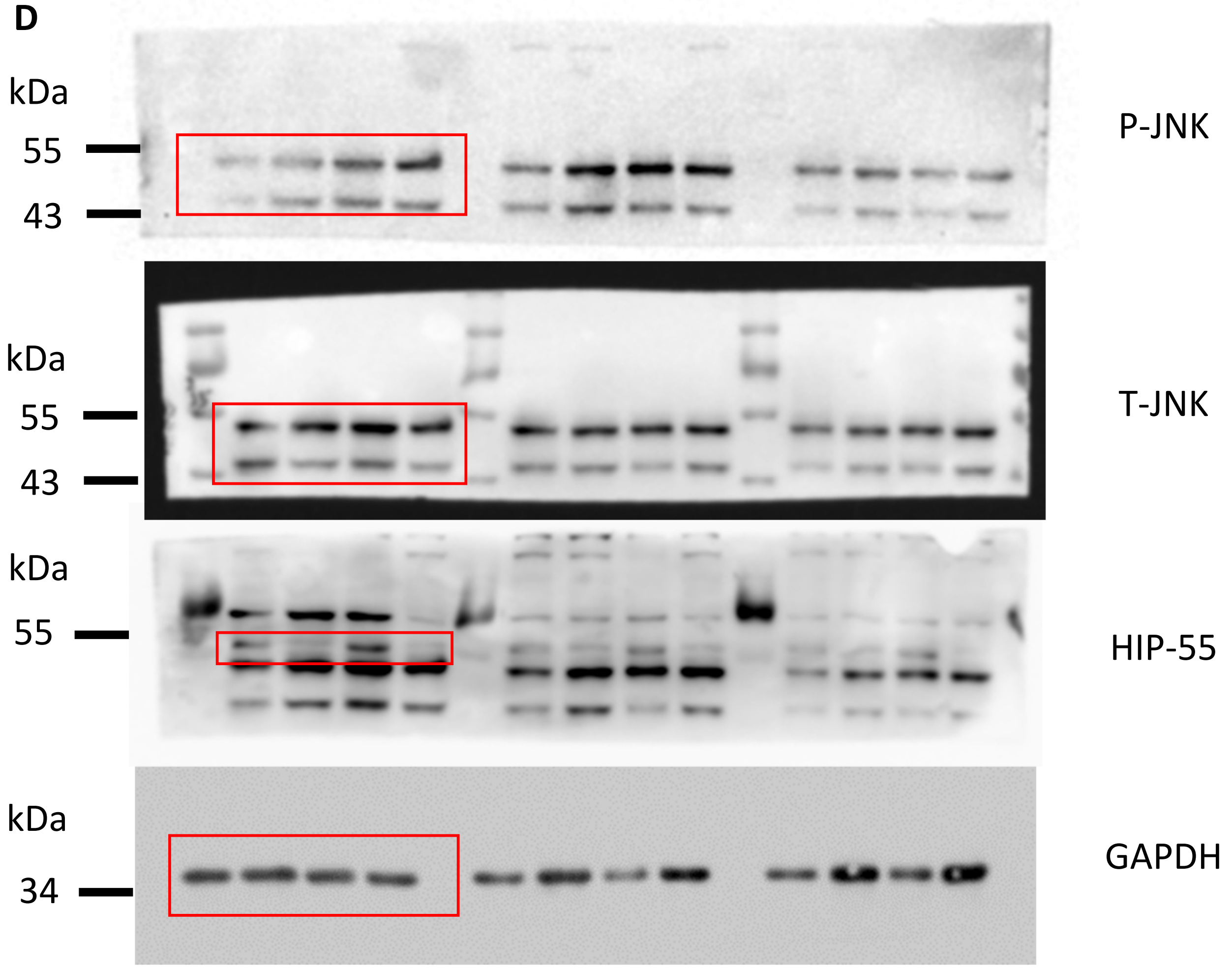

E

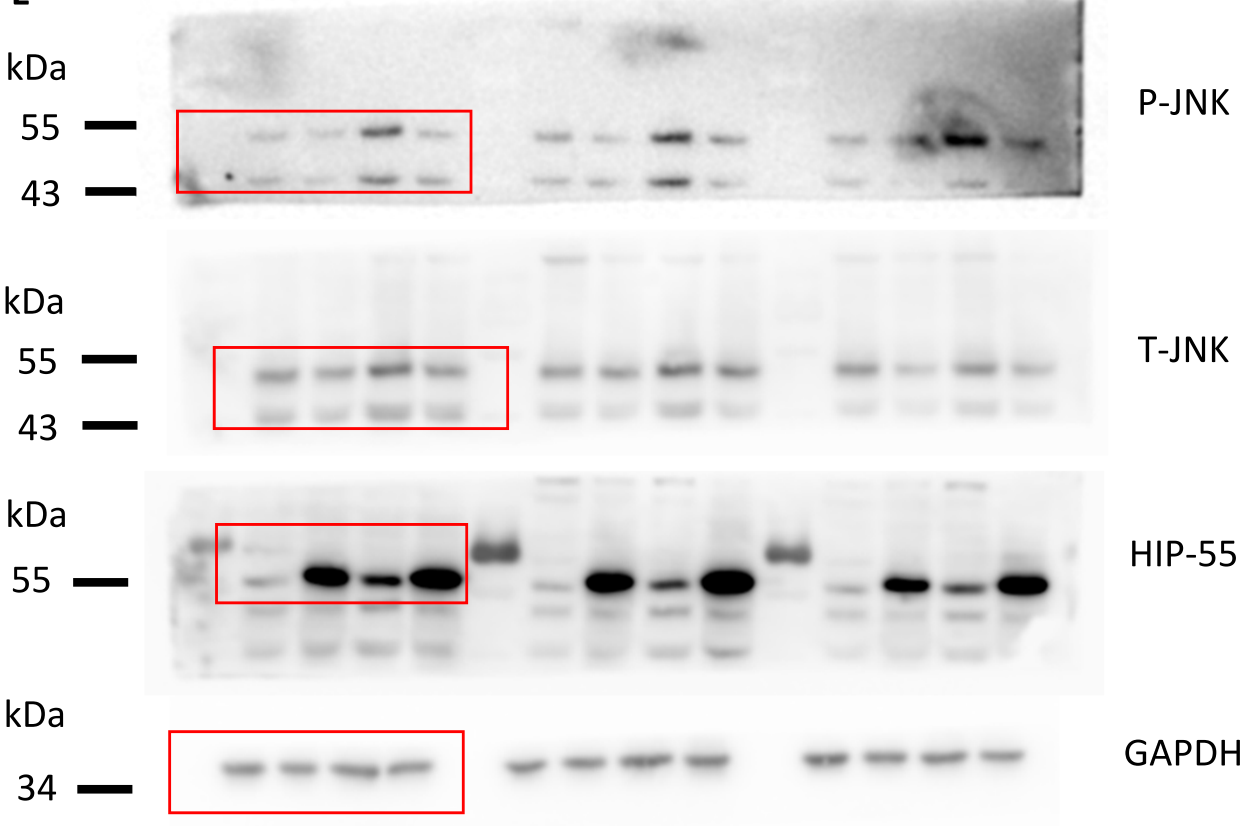

Uncropped images from Western blots

Uncropped images for Fig. 5

F

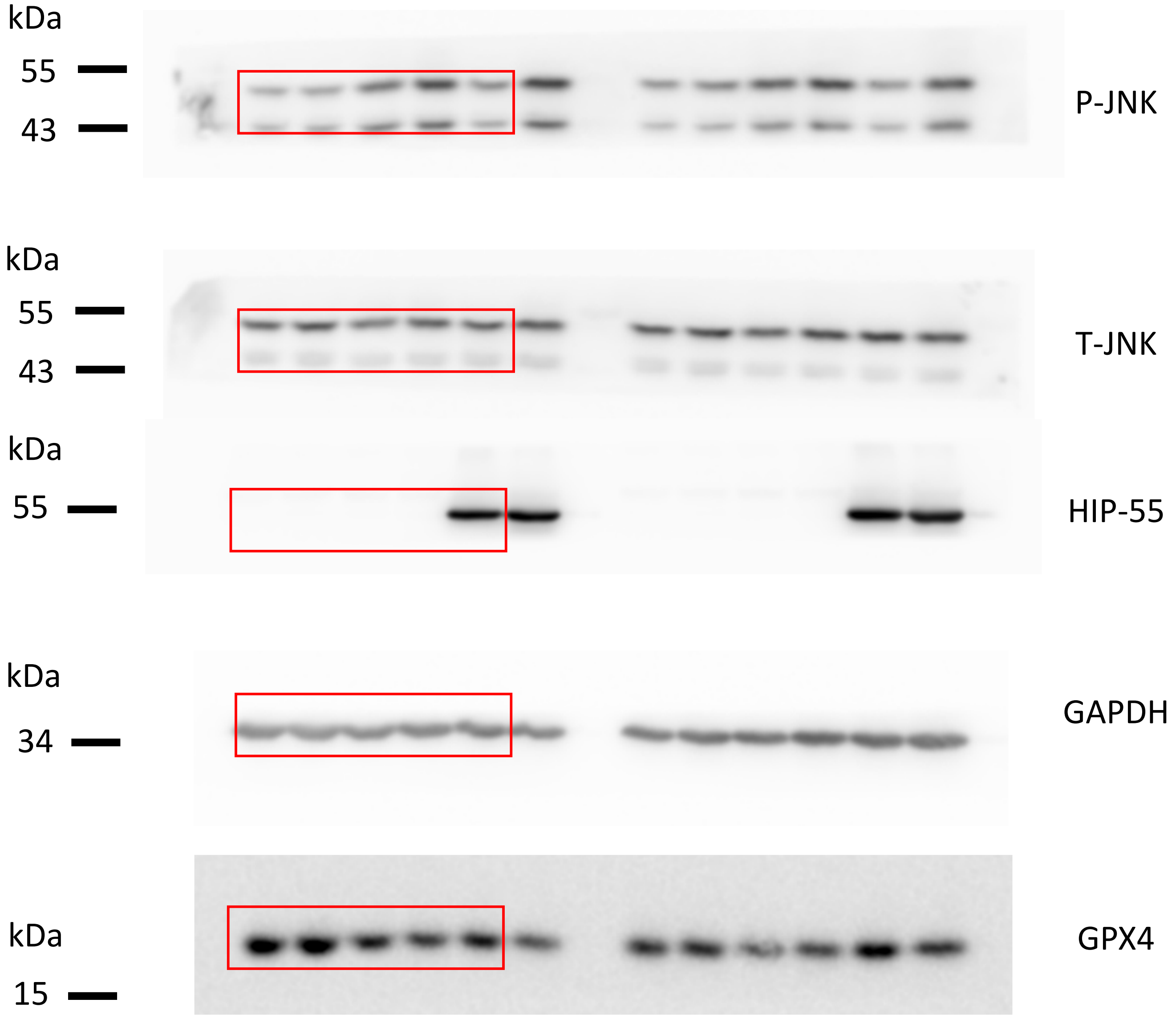

Uncropped images from Western blots

Uncropped images for Fig. 6

A

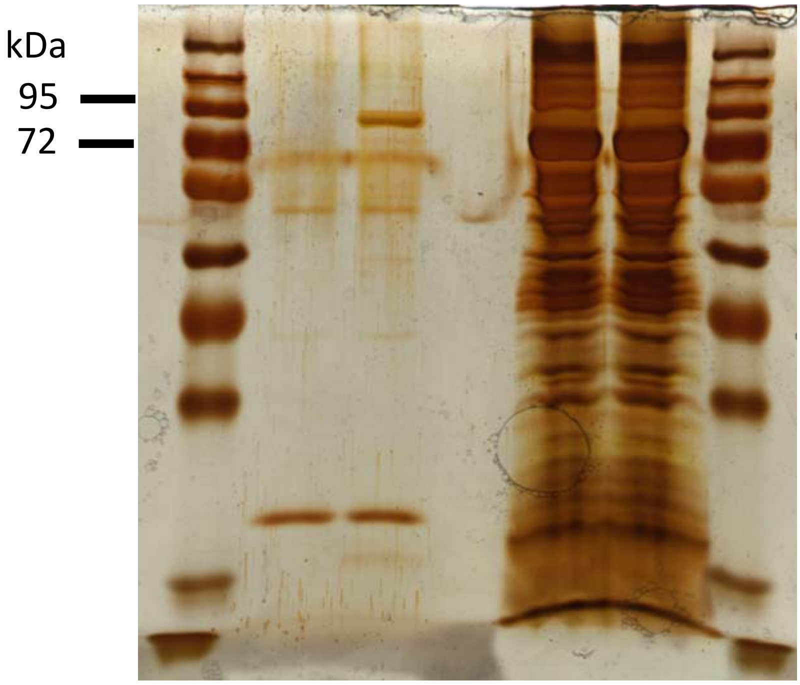

C

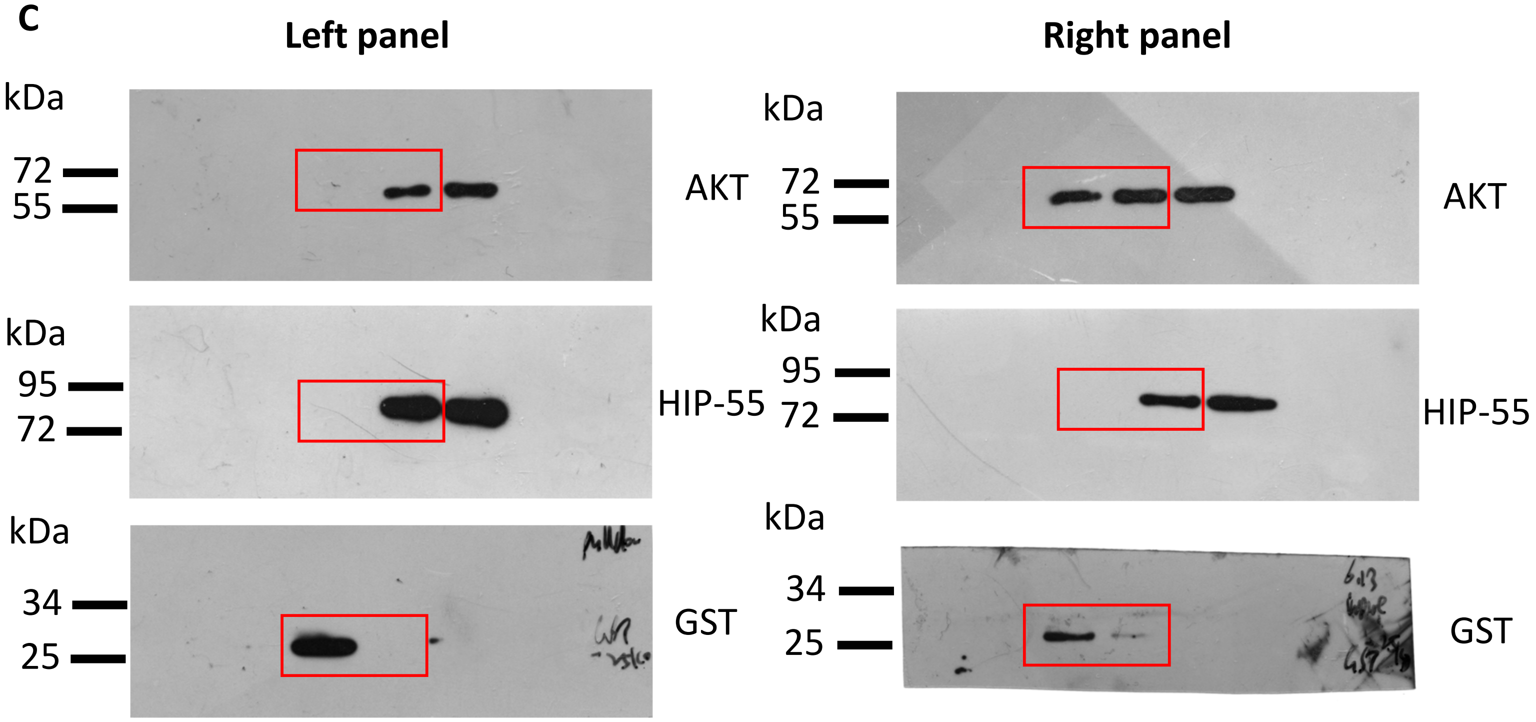

Uncropped images from Western blots

Uncropped images for Fig. 6

D

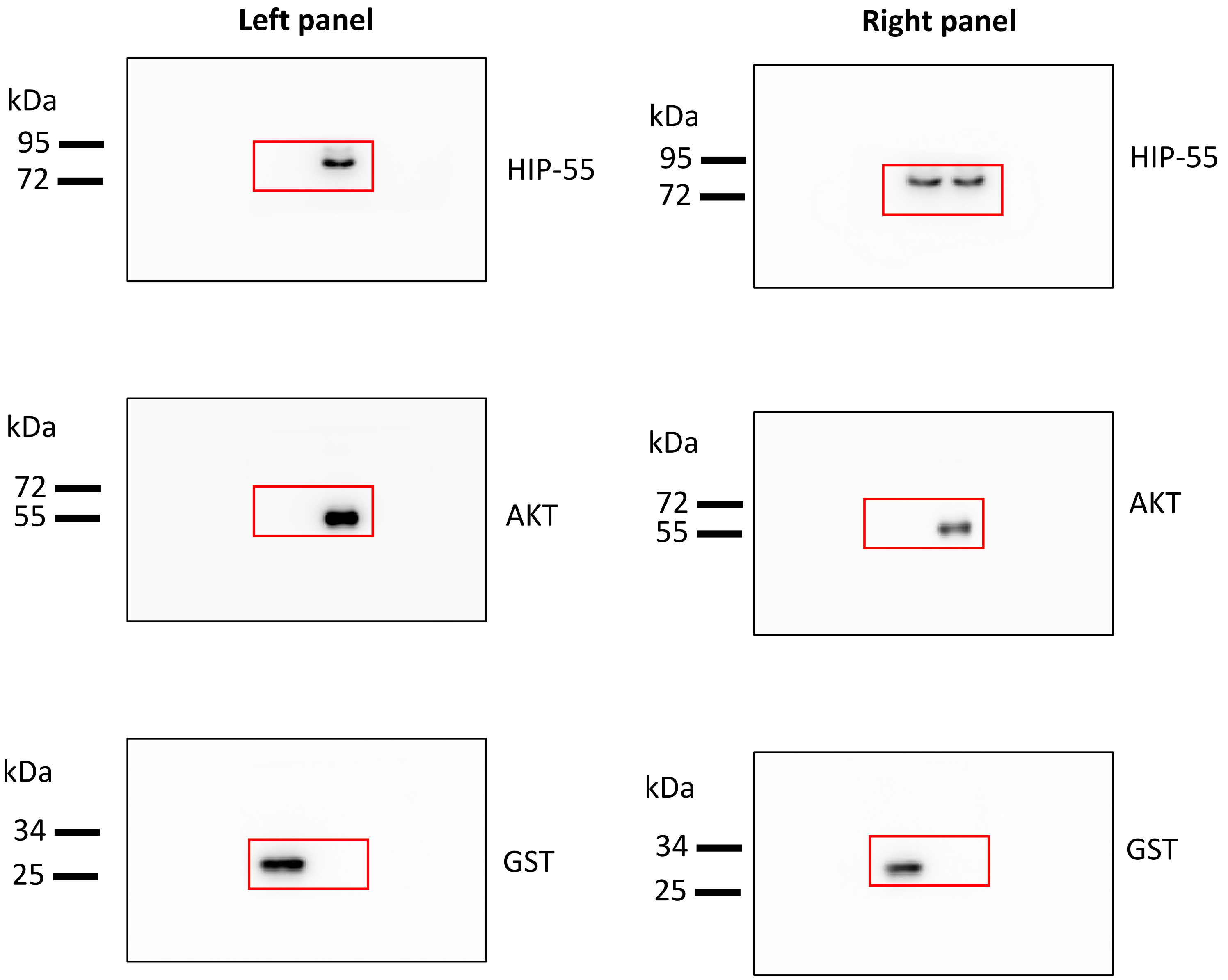

E

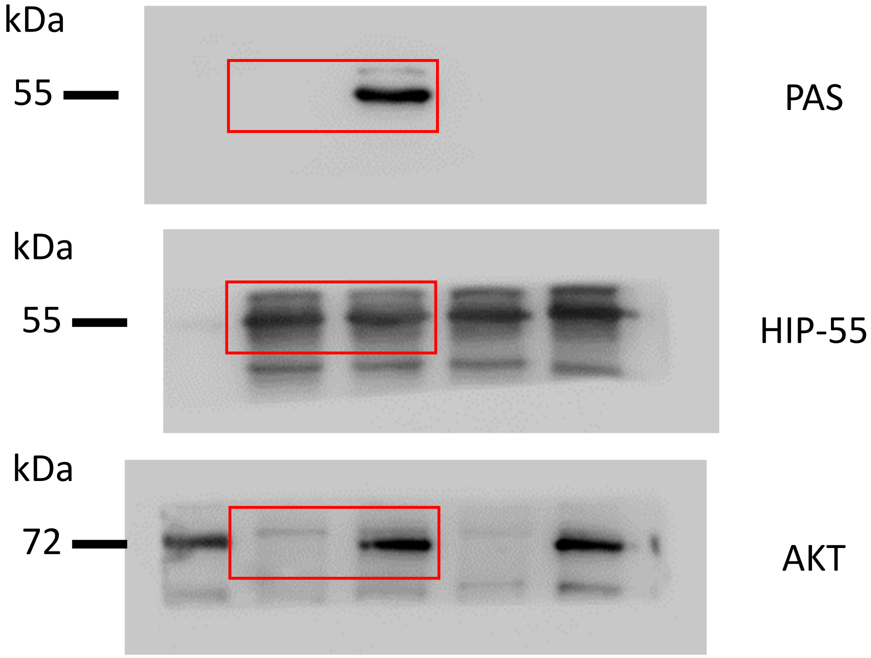

Uncropped images from Western blots

Uncropped images for Fig. 6

F

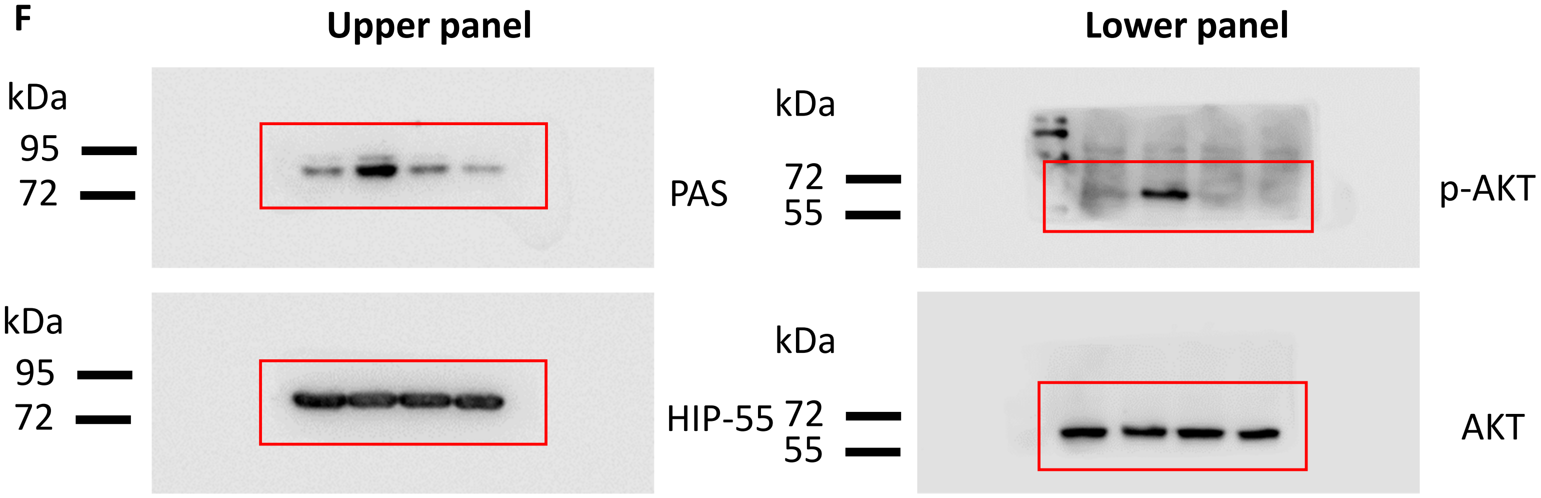

G

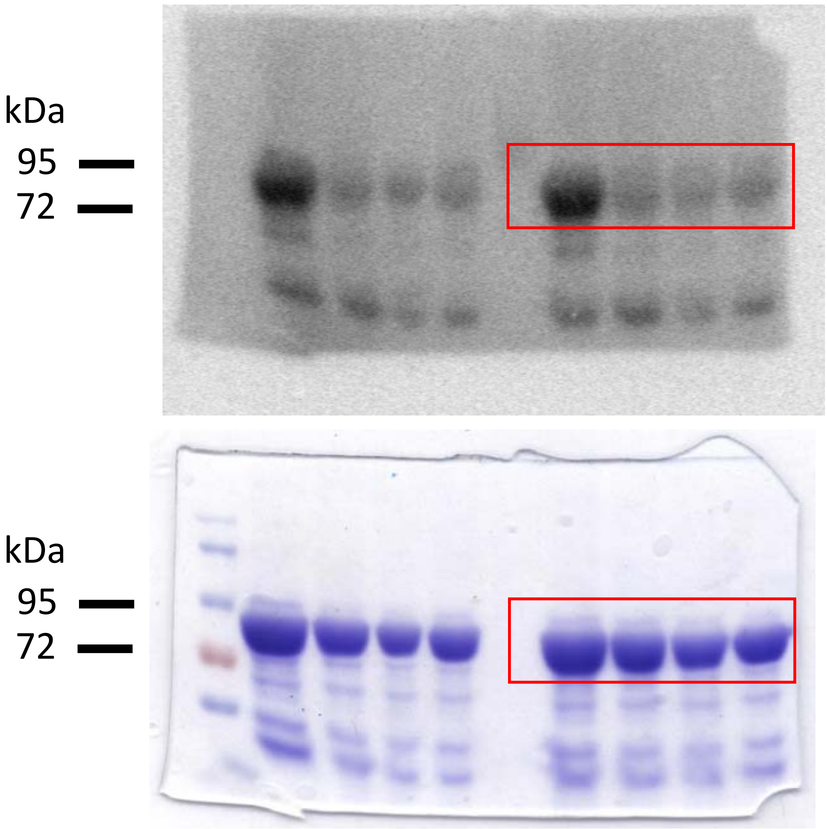

Uncropped images from Western blots

Uncropped images for Fig. 6

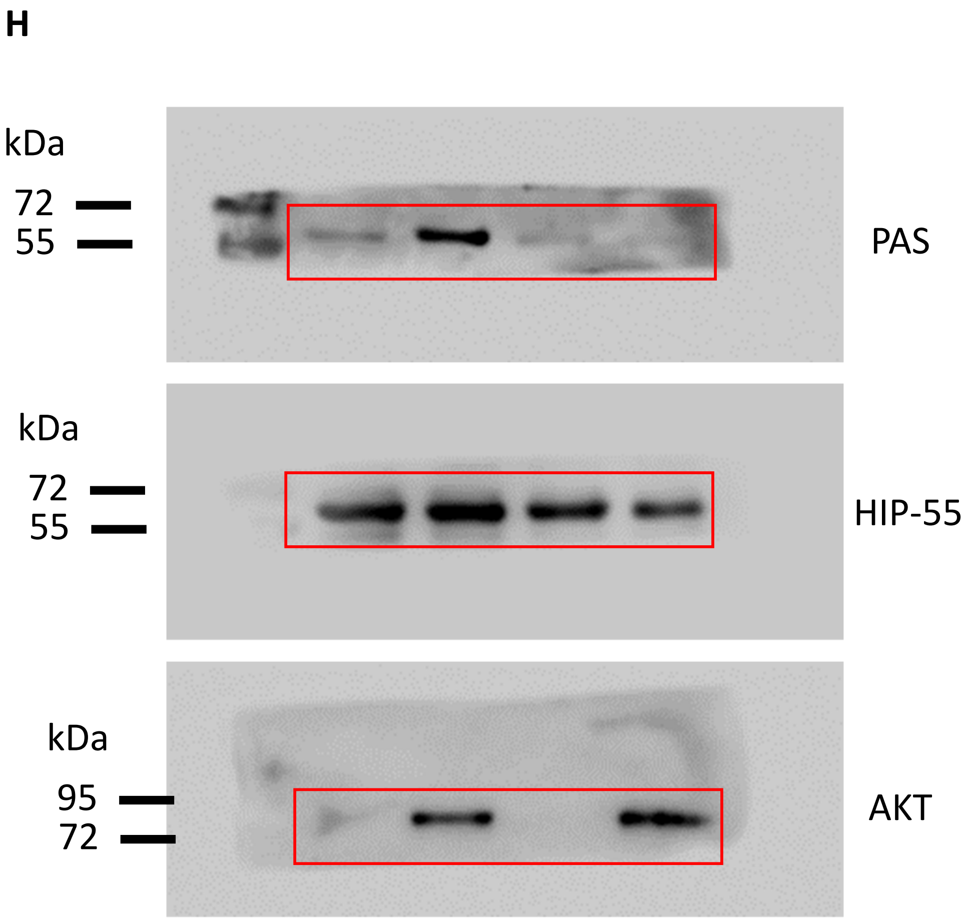

Uncropped images from Western blots

Uncropped images for Fig. 6

I

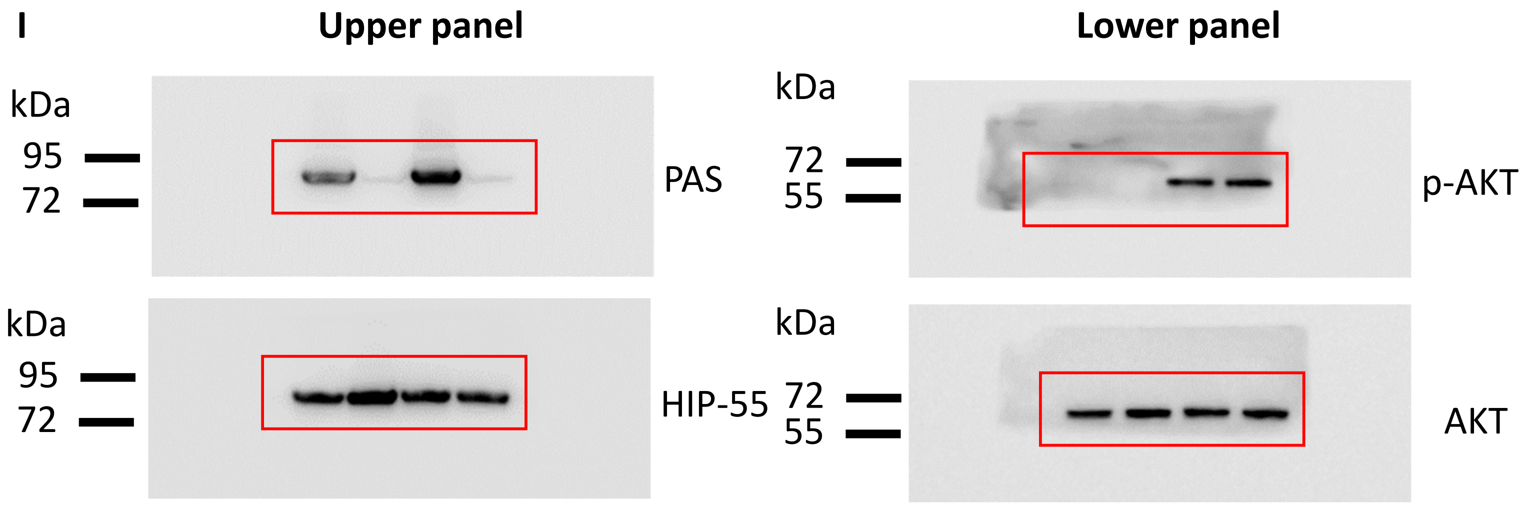

J

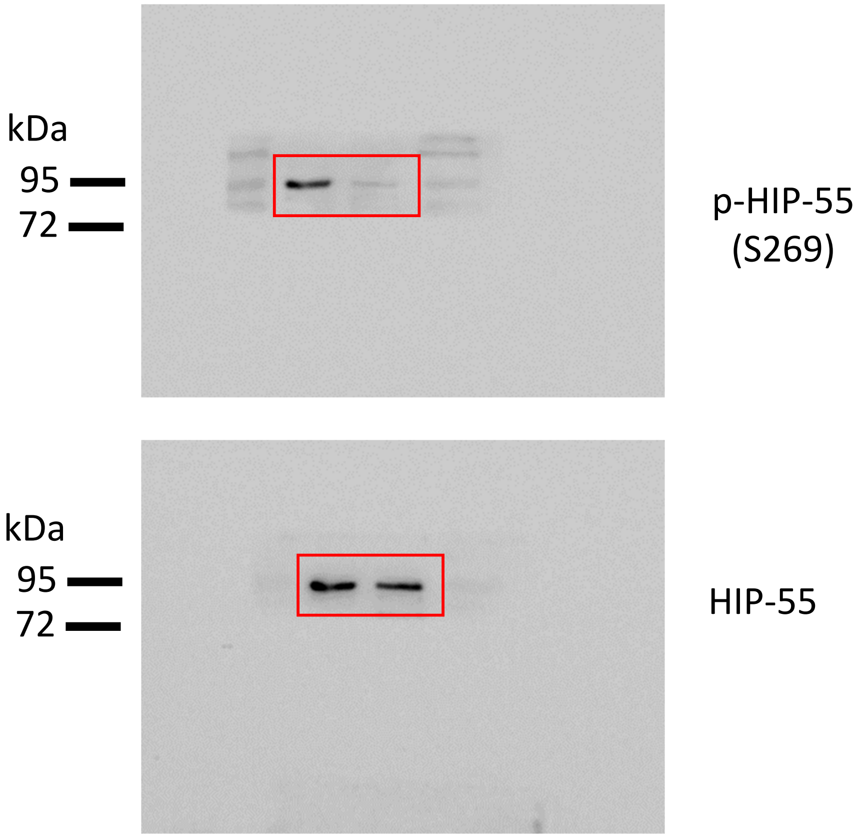

Uncropped images from Western blots

Uncropped images for Fig. 6

K

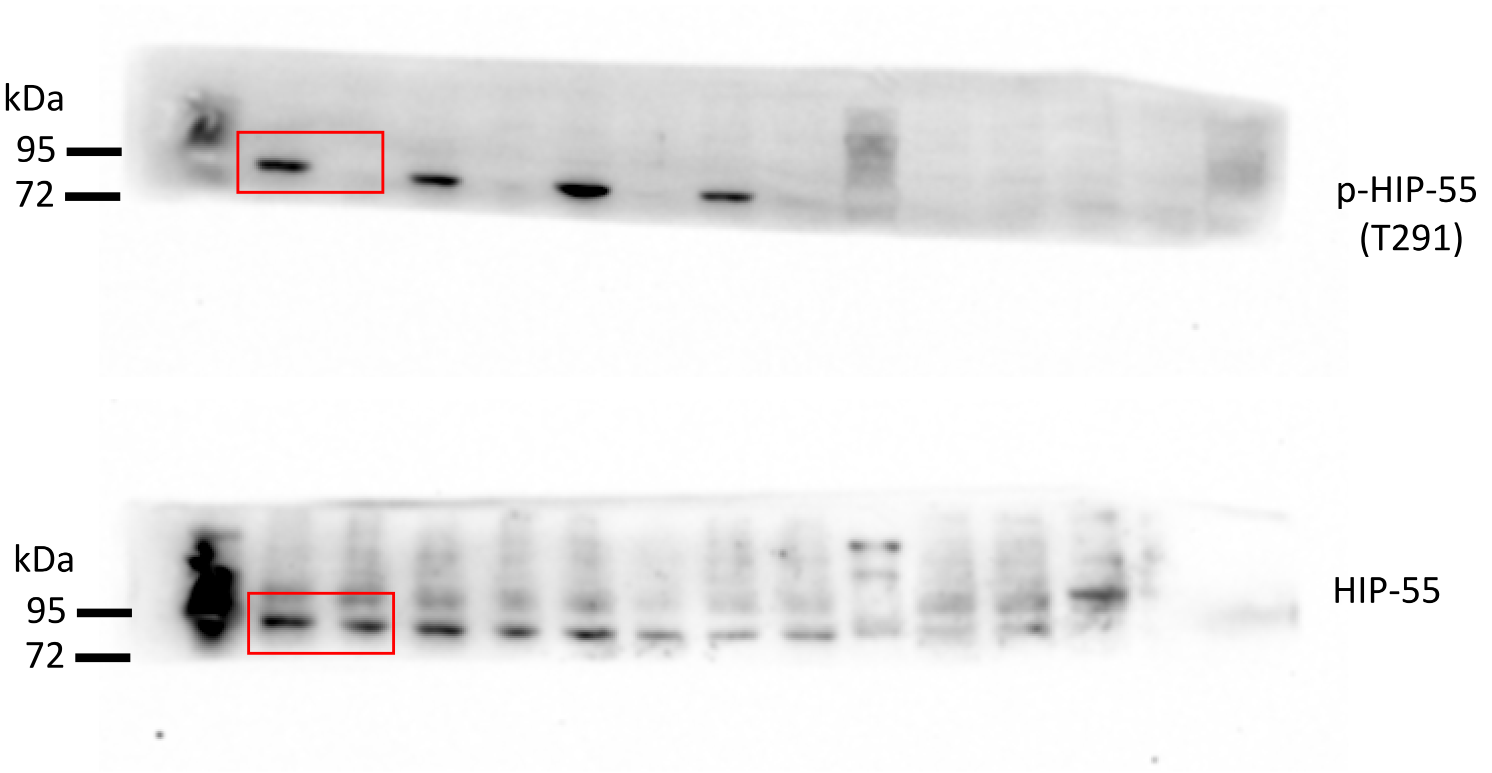

L

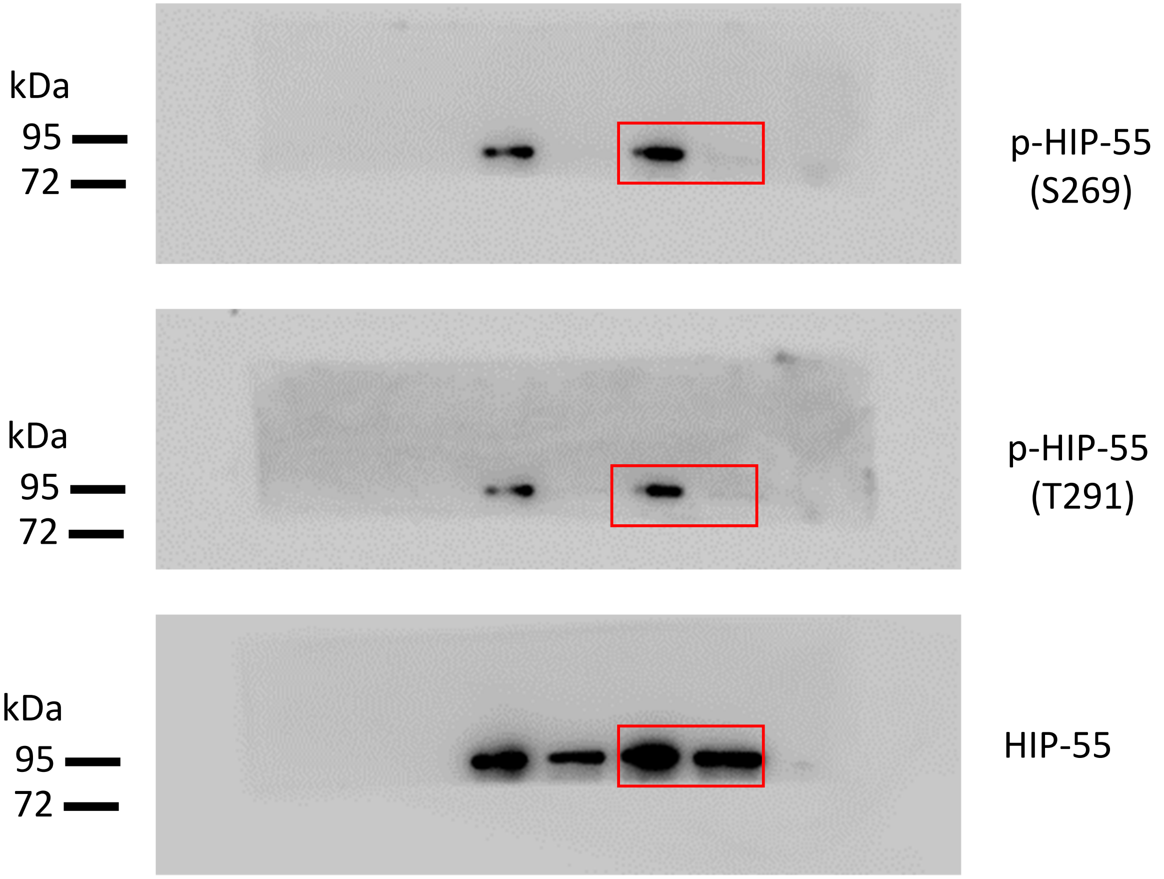

Uncropped images from Western blots

Uncropped images for Fig. 6

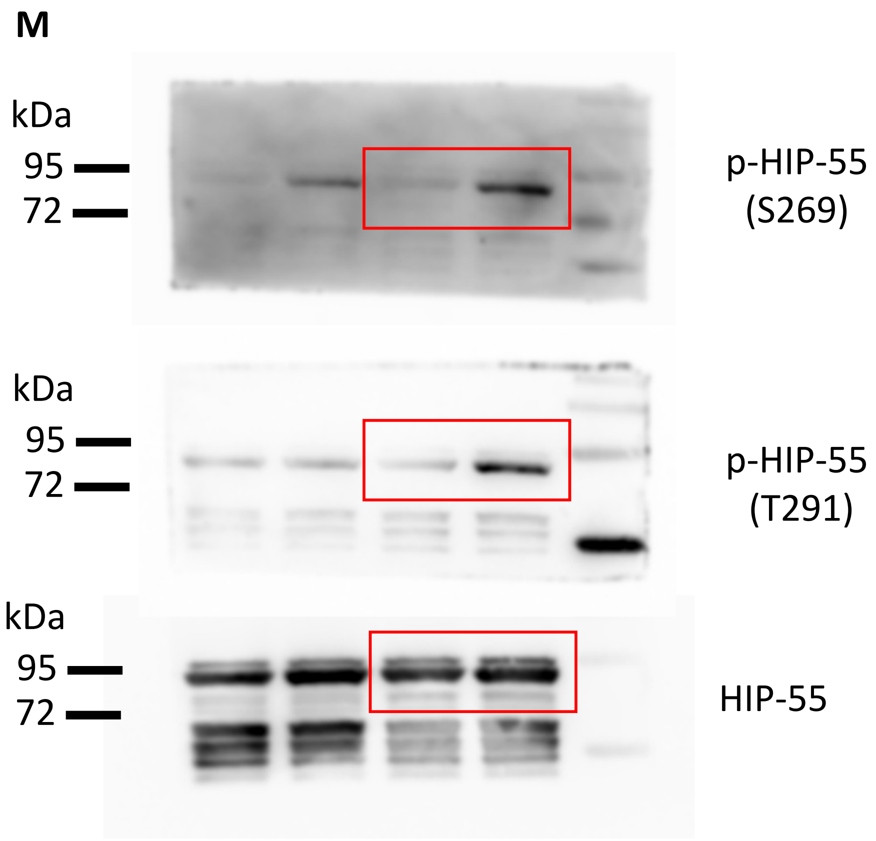

Uncropped images from Western blots

Uncropped images for Fig. 6

N

Upper panel

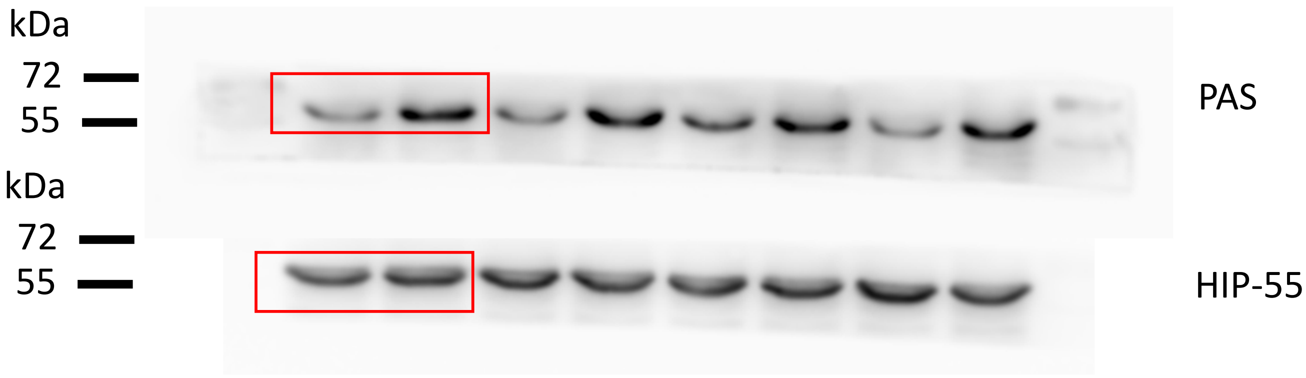

Lower panel

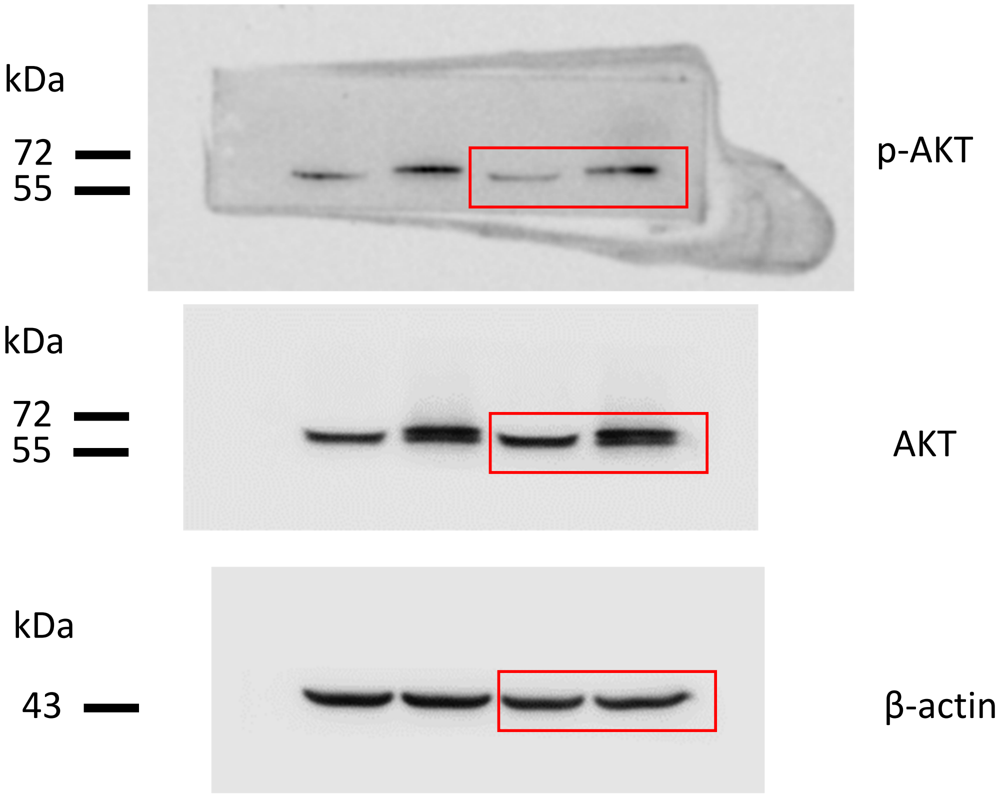

Uncropped images from Western blots

Uncropped images for Fig. 6

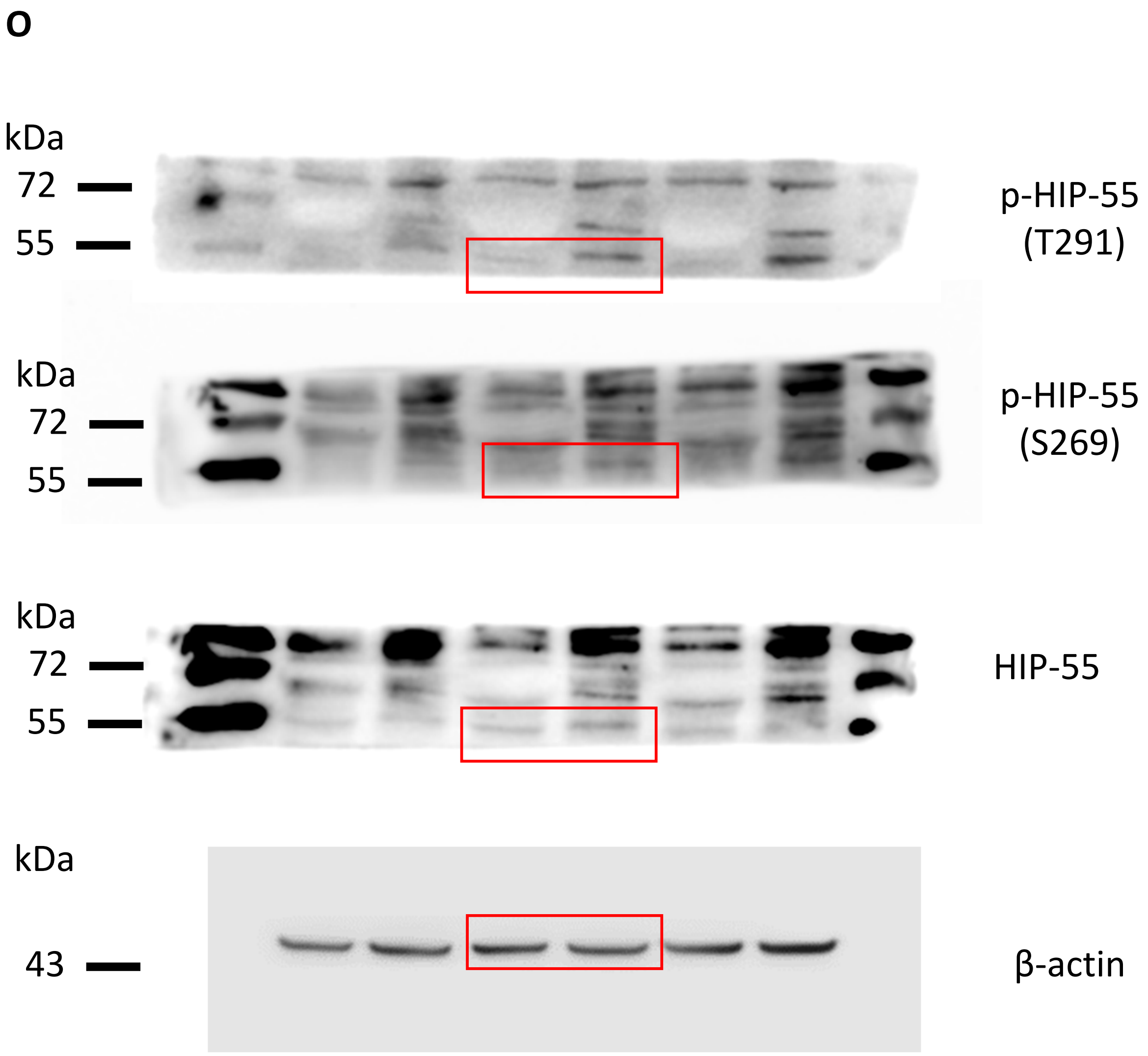

Uncropped images from Western blots

Uncropped images for Fig. 7

A

Upper panel

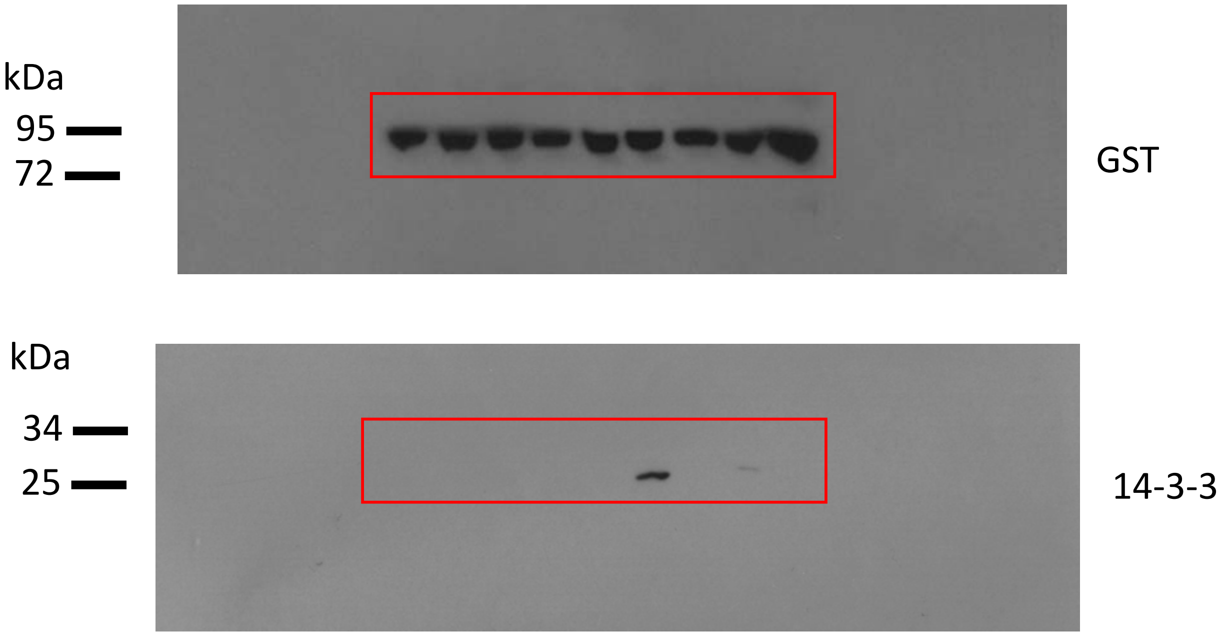

Lower panel

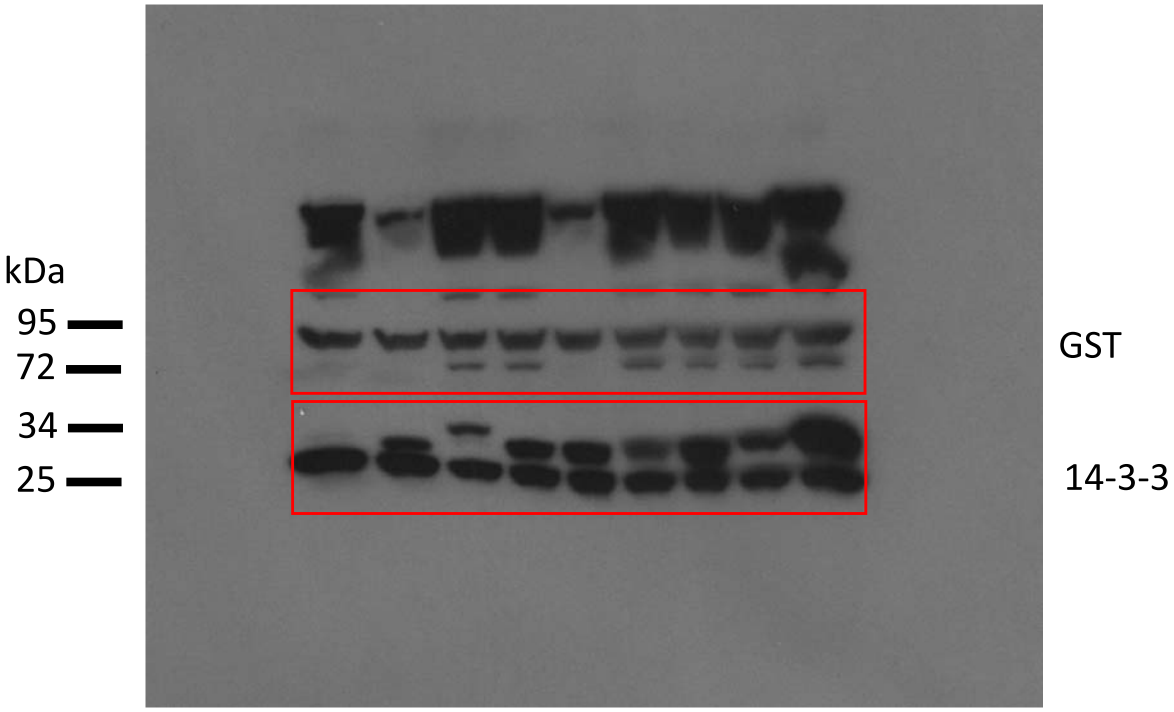

Uncropped images from Western blots

Uncropped images for Fig. 7

C

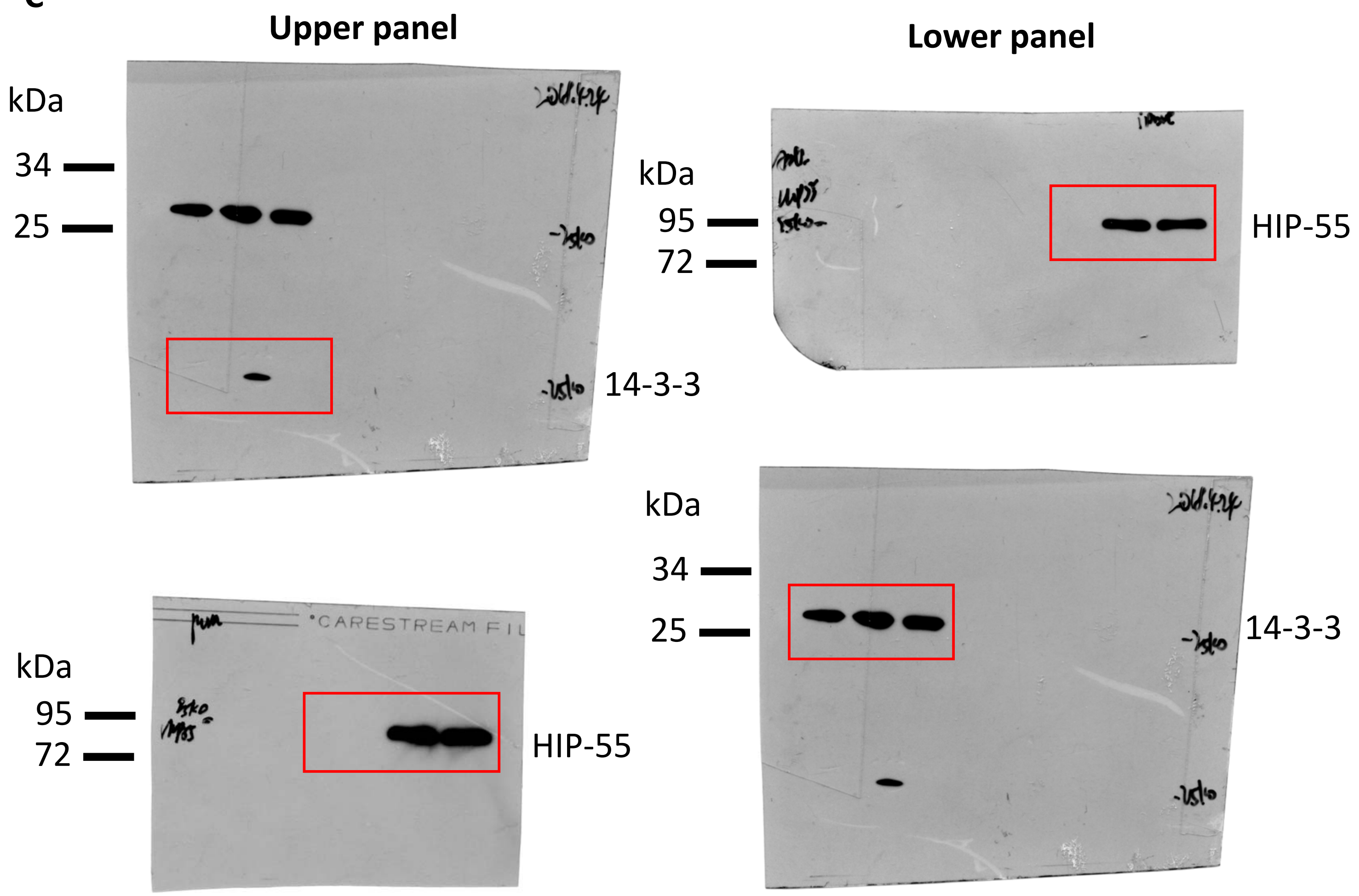

D

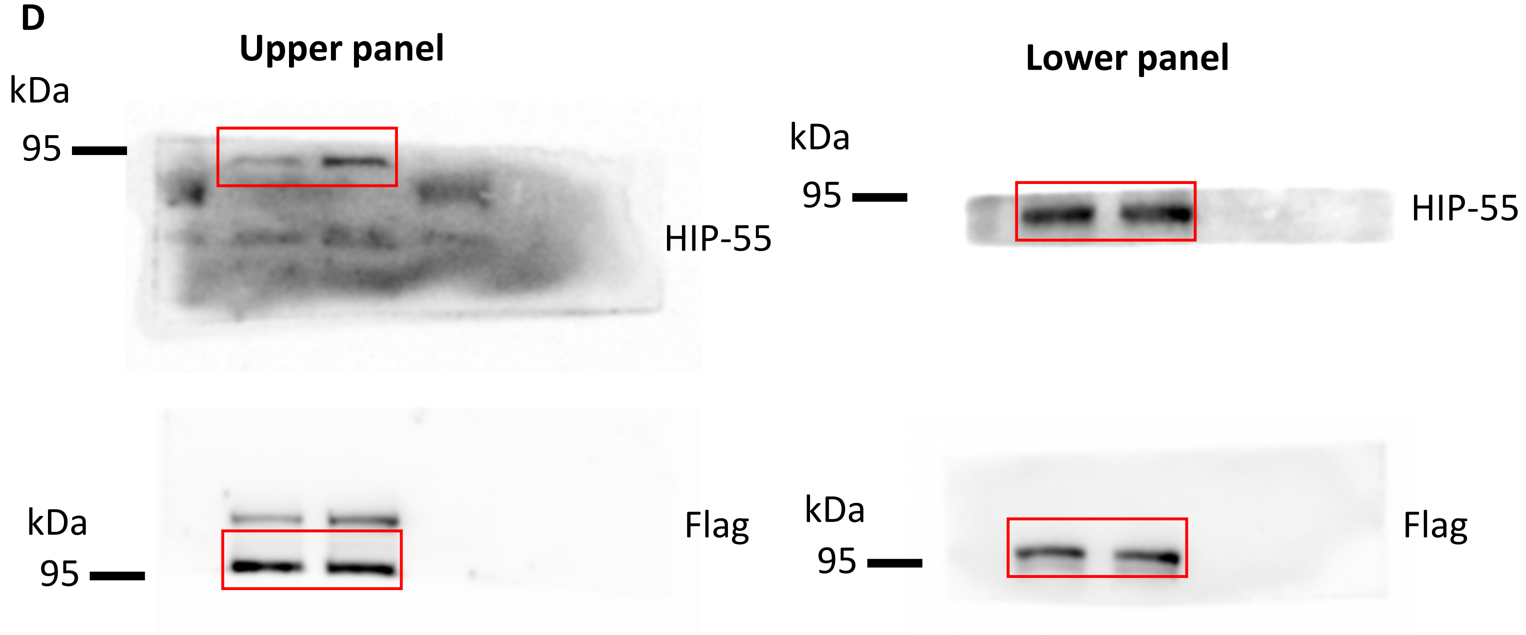

Uncropped images from Western blots

Uncropped images for Fig. 7

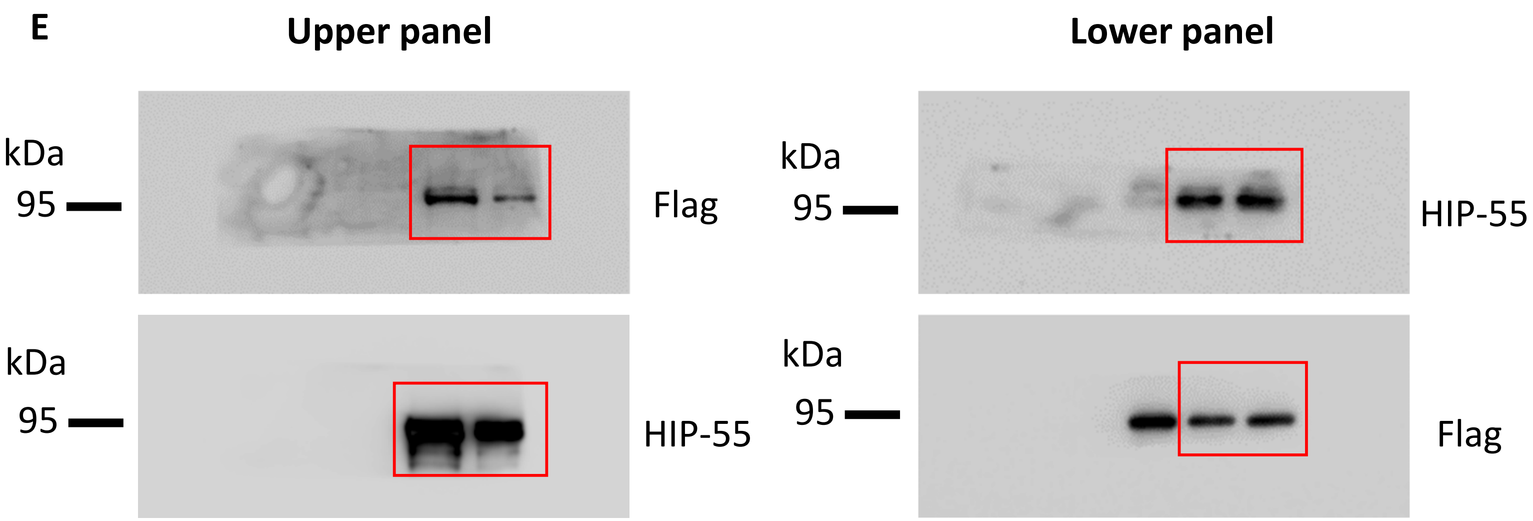

Uncropped images from Western blots

Uncropped images for Fig. 7

I

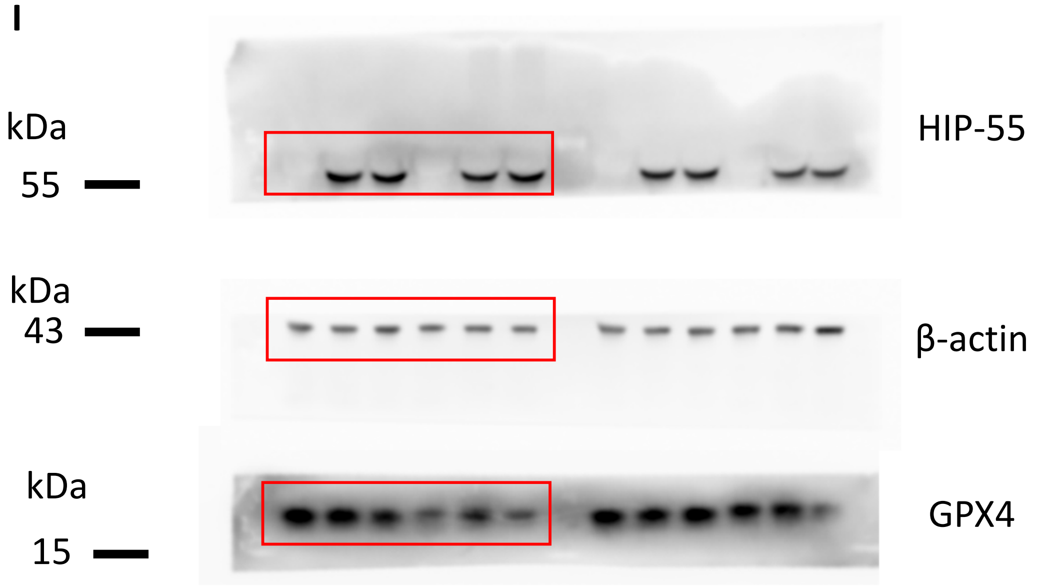

J

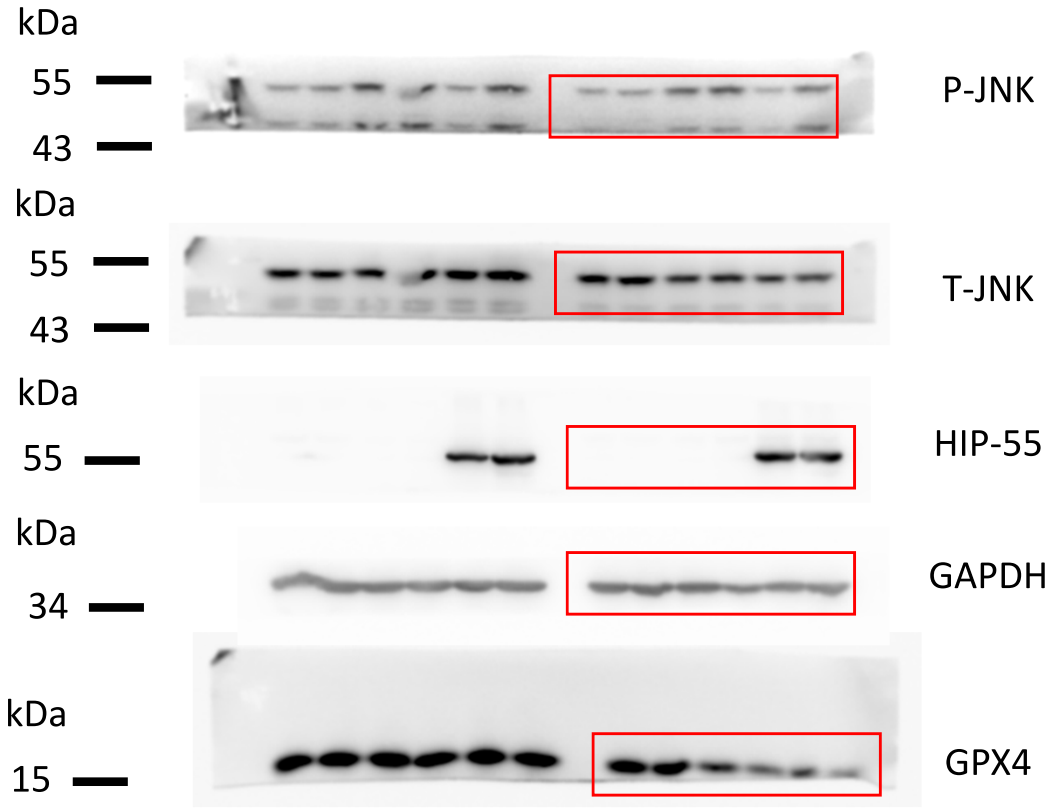

Uncropped images from Western blots

Uncropped images for Fig. 8

A

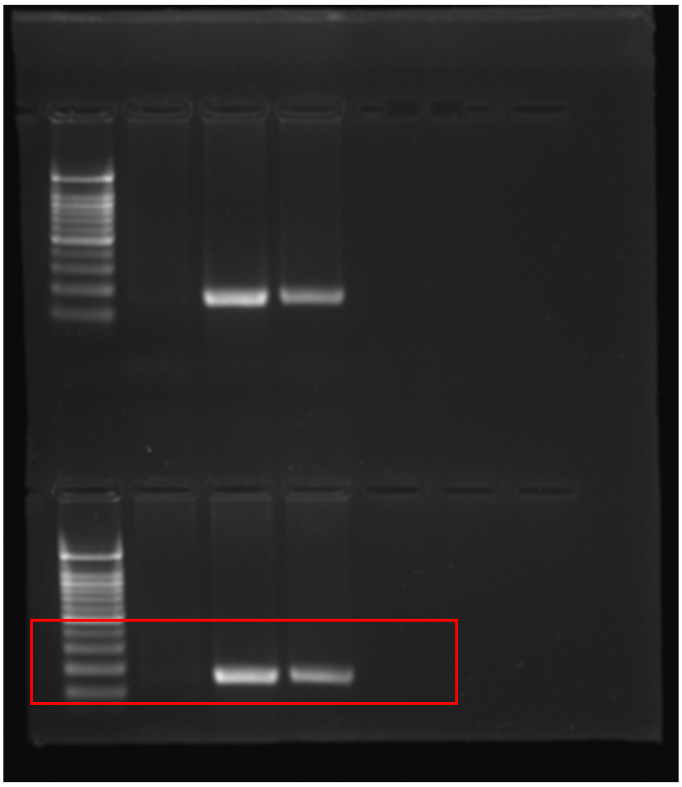

B

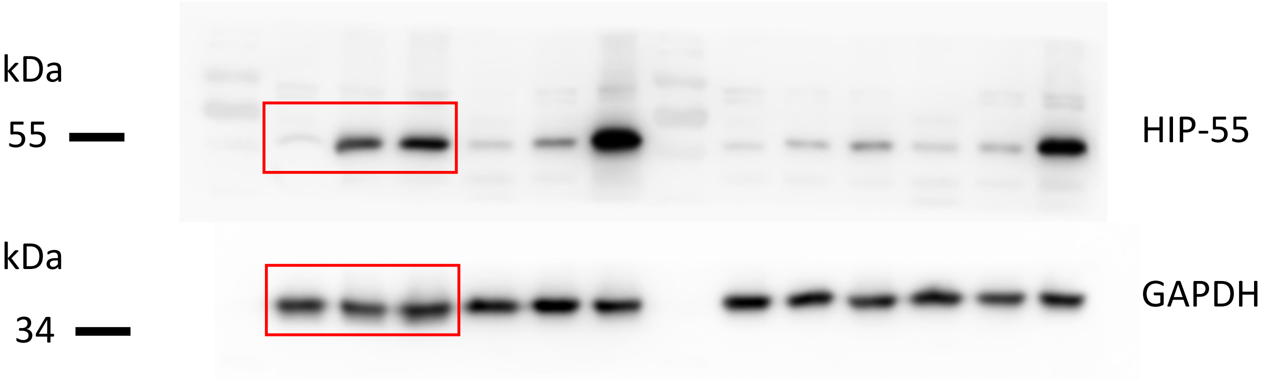

Uncropped images from Western blots

Uncropped images for Fig. 8

C

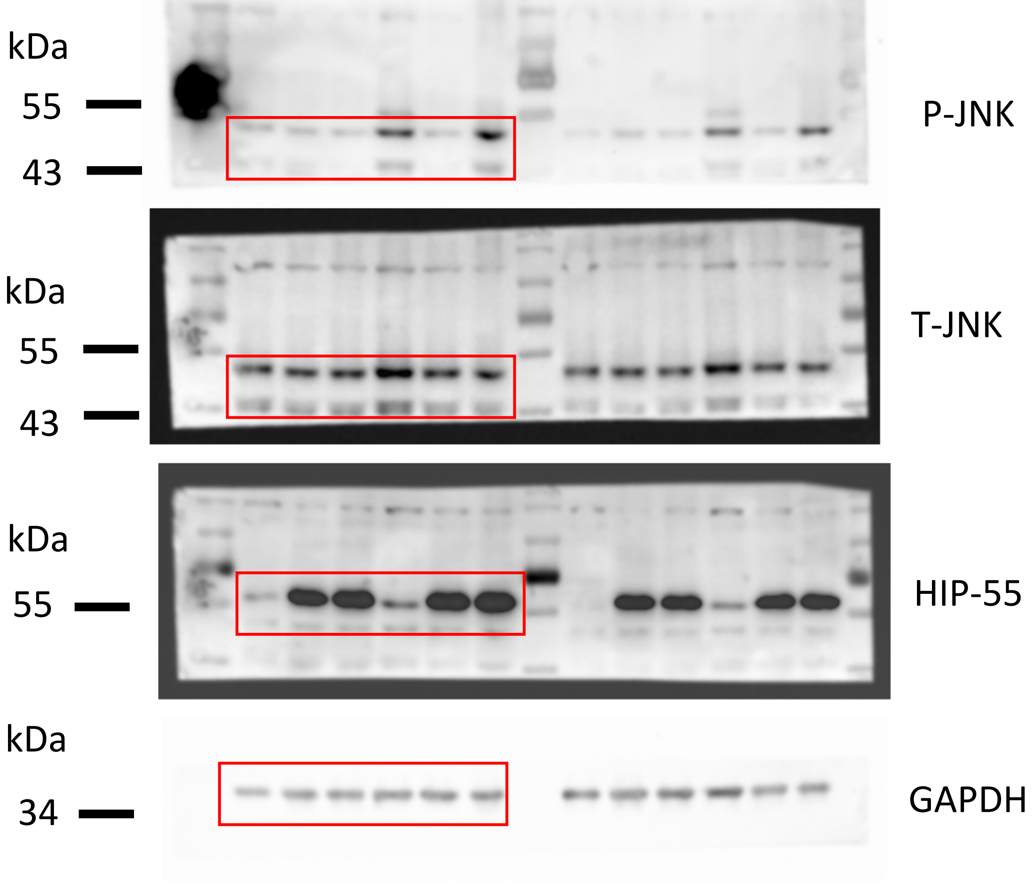

D

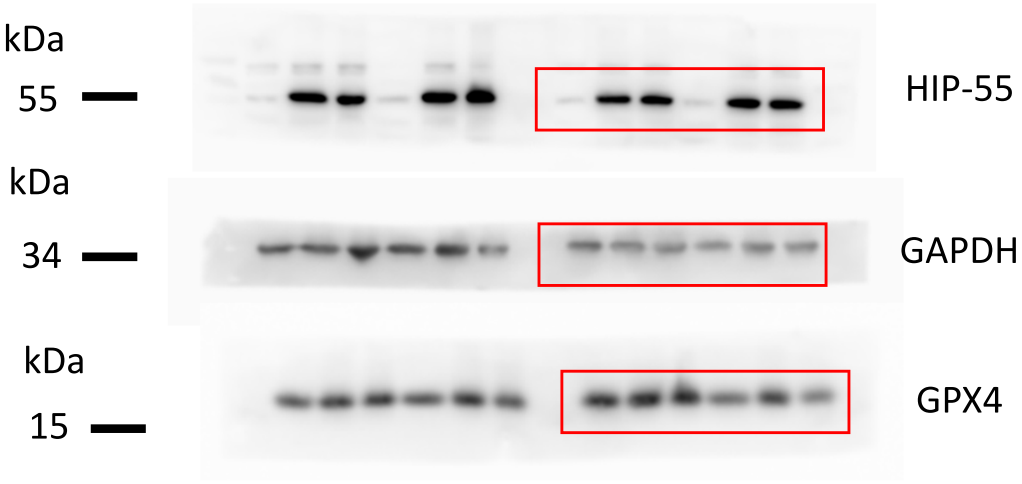

Supplement: Supplementary file 1 — Uncropped original western blots [file 41418_2022_1110_MOESM1_ESM.pdf]
