# Supplementary material for: Adaptor protein HIP-55-mediated signalosome protects against ferroptosis in myocardial infarction
Source: Cell Death Differ. 2023 Jan 13;30(3):825–38. doi: 10.1038/s41418-022-01110-z (PMC9984488; doi:10.1038/s41418-022-01110-z)
Supplement: Supplementary file 3 — CDD-author-contribution-form [file 41418_2022_1110_MOESM3_ESM.pdf]

DECLARATION OF CONTRIBUTIONS TO ARTICLE

ADMC

Manuscript Number: CDD-22-2138

Journal Name: *Cell Death & Differentiation* (the 'Journal')

Proposed Title of the Contribution:

Adaptor protein HIP-55-mediated signalosome protects against ferroptosis in myocardial infarction (the 'Contribution)

Author(s):

Yunqi Jiang, Yuhui Qiao, Dan He, Aiju Tian, Zijian Li (the 'Authors')

For all *CDD* articles, each person named as an author in the published version must be able to show he or she has contributed substantially to the article.

Authorship credit should be based on 1) substantial contributions to conception and design, acquisition of data, or analysis and interpretation of data; 2) drafting the article or revising it critically for important intellectual content; and 3) final approval of the version to be published. Authors should meet conditions 1, 2 and 3.

Any person who cannot be shown to have made a substantial contribution to the article cannot be listed as an author in the final version. The name of any person who is deemed to have made a minor contribution can, however, appear in the Acknowledgments section of the article.

Please complete the table below to indicate the contributions of all named authors to the manuscript.

Author Full Name:	Specification of Contribution to the Manuscript:
Zijian Li	Conceived and designed the project, wrote and revised the manuscript.
Yunqi Jiang	Designed this study, performed the experiments and analyzed the data, wrote and revised the manuscript.
Yuhui Qiao	Performed the myocardial infarction model experiments and analyzed the data.
Dan He	Performed the myocardial infarction model experiments and analyzed the data.
Aiju Tian	Performed the myocardial infarction model experiments and analyzed the data.

Please complete the table below to indicate the contributions of all named authors to the figures.

Figure 1:

Yunqi Jiang performed A-D; Yuhui Qiao performed E-F.

Figure 2:

Yunqi Jiang performed A-B; Yunqi Jiang, Yuhui Qiao, Dan He and Aiju Tian performed C; Yunqi Jiang and Yuhui Qiao performed D-E.

Figure 3:

Yunqi Jiang performed A-B; Yunqi Jiang, Yuhui Qiao, Dan He and Aiju Tian performed C; Yunqi Jiang and Yuhui Qiao performed D-E.

Figure 4:

Yunqi Jiang performed all the data.

Figure 5:

Yunqi Jiang performed all the data.

Figure 6:

Zijian Li performed G; Yunqi Jiang performed all the rest of data.

Figure 7: Zijian Li performed A; Yunqi Jiang performed all the rest of data.

Figure 8: Yunqi Jiang performed A-G; Yunqi Jiang, Yuhui Qiao, Dan He and Aiju Tian performed H; Yunqi Jiang and Yuhui Qiao performed I-J; Yunqi Jiang performed K.

Signed for and on behalf of the Author(s):	Print Name:	Date:
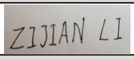	Zijian Li	November 16, 2022
